# Defects in intracellular trafficking of fungal cell wall synthases lead to aberrant host immune recognition

**DOI:** 10.1371/journal.ppat.1007126

**Published:** 2018-06-04

**Authors:** Shannon K. Esher, Kyla S. Ost, Maria A. Kohlbrenner, Kaila M. Pianalto, Calla L. Telzrow, Althea Campuzano, Connie B. Nichols, Carol Munro, Floyd L. Wormley, J. Andrew Alspaugh

**Affiliations:** 1 Departments of Molecular Genetics and Microbiology/Medicine, Duke University School of Medicine, Durham, NC, United States of America; 2 Department of Biology, University of Texas at San Antonio, San Antonio, Texas, United States of America; 3 MRC Centre for Medical Mycology, University of Aberdeen, Institute of Medical Sciences, Foresterhill, Aberdeen, United Kingdom; University of Birmingham, UNITED KINGDOM

## Abstract

The human fungal pathogen, *Cryptococcus neoformans*, dramatically alters its cell wall, both in size and composition, upon entering the host. This cell wall remodeling is essential for host immune avoidance by this pathogen. In a genetic screen for mutants with changes in their cell wall, we identified a novel protein, Mar1, that controls cell wall organization and immune evasion. Through phenotypic studies of a loss-of-function strain, we have demonstrated that the *mar1Δ* mutant has an aberrant cell surface and a defect in polysaccharide capsule attachment, resulting in attenuated virulence. Furthermore, the *mar1Δ* mutant displays increased staining for exposed cell wall chitin and chitosan when the cells are grown in host-like tissue culture conditions. However, HPLC analysis of whole cell walls and RT-PCR analysis of cell wall synthase genes demonstrated that this increased chitin exposure is likely due to decreased levels of glucans and mannans in the outer cell wall layers. We observed that the Mar1 protein differentially localizes to cellular membranes in a condition dependent manner, and we have further shown that the *mar1Δ* mutant displays defects in intracellular trafficking, resulting in a mislocalization of the β-glucan synthase catalytic subunit, Fks1. These cell surface changes influence the host-pathogen interaction, resulting in increased macrophage activation to microbial challenge *in vitro*. We established that several host innate immune signaling proteins are required for the observed macrophage activation, including the Card9 and MyD88 adaptor proteins, as well as the Dectin-1 and TLR2 pattern recognition receptors. These studies explore novel mechanisms by which a microbial pathogen regulates its cell surface in response to the host, as well as how dysregulation of this adaptive response leads to defective immune avoidance.

## Author summary

Disease causing microorganisms have adapted many strategies to avoid host immune detection in order to facilitate their survival. Pathogenic fungi alter their cell surface in order to mask immune stimulatory epitopes in their cell wall. We have identified a novel protein involved in this host-induced cell wall remodeling process in *Cryptococcus neoformans*. We have created a mutant strain with a targeted deletion of this gene and demonstrated that this protein is involved in intracellular trafficking of the β-(1,3)-glucan synthase enzyme and is differentially localized in response to host-like conditions. In the absence of this protein, the cell wall has decreased levels of β-glucan and increased exposure of chitooligomers. Importantly, improper cell wall maintenance by this mutant leads to a hyper-immunostimulatory fungal cell surface that induces increased macrophage activation. We show that this activation is dependent on several pattern recognition receptors previously demonstrated to be important for fungal pathogenesis.

## Introduction

The microbial surface is the first point of contact for interactions with the innate immune system, representing the site at which an infected host might recognize a microbe as a potential pathogen. This recognition is achieved through host pattern recognition receptors (PRRs) that distinguish specific pathogen-associated molecular patterns (PAMPs) on microbial surfaces, directing downstream signaling events that ultimately lead to the initiation of an immune response. The fungal cell wall is a dynamic structure composed of a complex matrix of polysaccharides including α- and β-glucans, mannoproteins (mannans), and chitin/chitosan. These fungal specific components have been shown by many groups to be recognized by host PRRs including Toll-like receptors (TLRs) and C-type lectin receptors (CLRs) [1,2]. Several fungi have developed strategies to mask their surfaces from immune detection. Examples include *Histoplasma capsulatum* cell wall α-(1,3)-glucan and *Aspergillus fumigatus* conidial RodA hydrophobin, which both serve to block exposure of the more immunogenic β-glucan molecule [3,4]. The fungal cell surface is also responsive to different environments, including various micro-environments within the infected host. For example, *Candida albicans* differentially exposes β-glucan in response to diverse host niches, drug treatments, and growth conditions, resulting in varying degrees of Dectin-1-mediated host responses [5,6].

The opportunistic human fungal pathogen *Cryptococcus neoformans* continues to be a significant health threat for immune compromised populations, particularly those with HIV/AIDS, among whom it causes over 175,000 deaths per year [7]. This ubiquitous fungus colonizes the lungs after inhalation from the environment. It can then disseminate to the central nervous system in immunocompromised individuals, where it causes life-threatening meningoencephalitis [8]. *C*. *neoformans* has developed several adaptations to avoid immune detection and to direct the host immune response in its favor. The polysaccharide capsule on the cell surface shields immunostimulatory cell wall components from host recognition. Additionally, secreted capsular material actively represses various immune responses [9]. *C*. *neoformans* cells can also grow to massive sizes, forming so-called titan cells in the setting of infection. These giant and hyper-encapsulated cells are unable to be engulfed by host immune cells, but instead they drive a non-protective immune response to *C*. *neoformans* leading to pathogen persistence [10–12].

Both capsule production and titan cell formation are induced in the host environment and involve significant cell wall remodeling. Previous work has shown that capsule polysaccharide likely attaches to the cell surface through interaction with α-(1,3)-glucan in the cell wall [13]. Titan cell walls are also thicker and more chitin-rich then normal sized cells [12]. In addition to these macromolecular cell surface changes, our lab has shown that *C*. *neoformans* actively remodels its cell wall in response to host pH signals in order to avoid immune detection [14–16]. We have recently demonstrated that aberrant chitooligomer exposure leads to a detrimental immune response [16]. Although few investigators have performed detailed cell wall analyses in *C*. *neoformans*, this structure appears to contain significantly more chitin and chitosan than that of other pathogenic species [17]. There is increasing evidence to support the concept of chitin as an immune modulatory molecule, and recent studies have highlighted the complexity of chitin recognition, indicating that the source and size of chitin molecules can differentially direct immune responses [18–20].

*C*. *neoformans* cell wall regulation, particularly in response to the host environment, remains incompletely defined. Our lab has previously identified cell surface properties that drive immune detection and through a previously published screen for cell wall regulators, we have identified a novel protein involved in this process [14,21,22]. In this study we explore the mechanism by which this protein, Mar1, regulates cell wall remodeling, and the implications of an aberrant cell wall architecture on host immune detection. We report that in the absence of Mar1, cells display a defect in capsule attachment, as well as increased exposure of cell wall chitin and chitosan. This concurs with our observation of decreased levels of glucans and mannans in the cell wall, decreased expression of α- and β-glucan synthases, and mislocalization of the β-glucan synthase, Fks1. While canonical secretion is intact in the *mar1Δ* mutant strain, general intracellular trafficking appears altered. We also report the localization of the Mar1 protein itself to cellular membranes and demonstrate differential localization in host-like tissue culture conditions. The implications of this cell wall remodeling defect include increased recognition and activation by innate immune cells and attenuated virulence. We further show that this innate immune recognition is dependent on the Card9 and MyD88 adaptor proteins and the cell surface receptors Dectin-1 and TLR2. In addition to highlighting intracellular processes involved in cell wall remodeling, these studies underscore the importance of proper cell wall regulation in host immune detection and broaden our understanding of the recognition of individual fungal cell wall components.

## Results

### Identification of a novel cell wall regulatory protein in *Cryptococcus neoformans*

The cell wall is the interface between microbial pathogens and host immune cells. To identify genes required for proper fungal cell wall homeostasis, we performed a random mutagenesis screen in the human fungal pathogen *Cryptococcus neoformans* using *Agrobacterium tumefaciens*-mediated transformation (AMT). Mutagenized strains were screened for phenotypes corresponding to cell wall changes, including growth impairment and dry colony morphology in alkaline conditions (pH 8) and sensitivity to elevated salt concentrations (1.5 M NaCl). We hypothesized that a subset of these cell wall mutants would also display alterations in the host-pathogen interaction. Preliminary results from this screen were previously reported [22].

**Fig 1:**
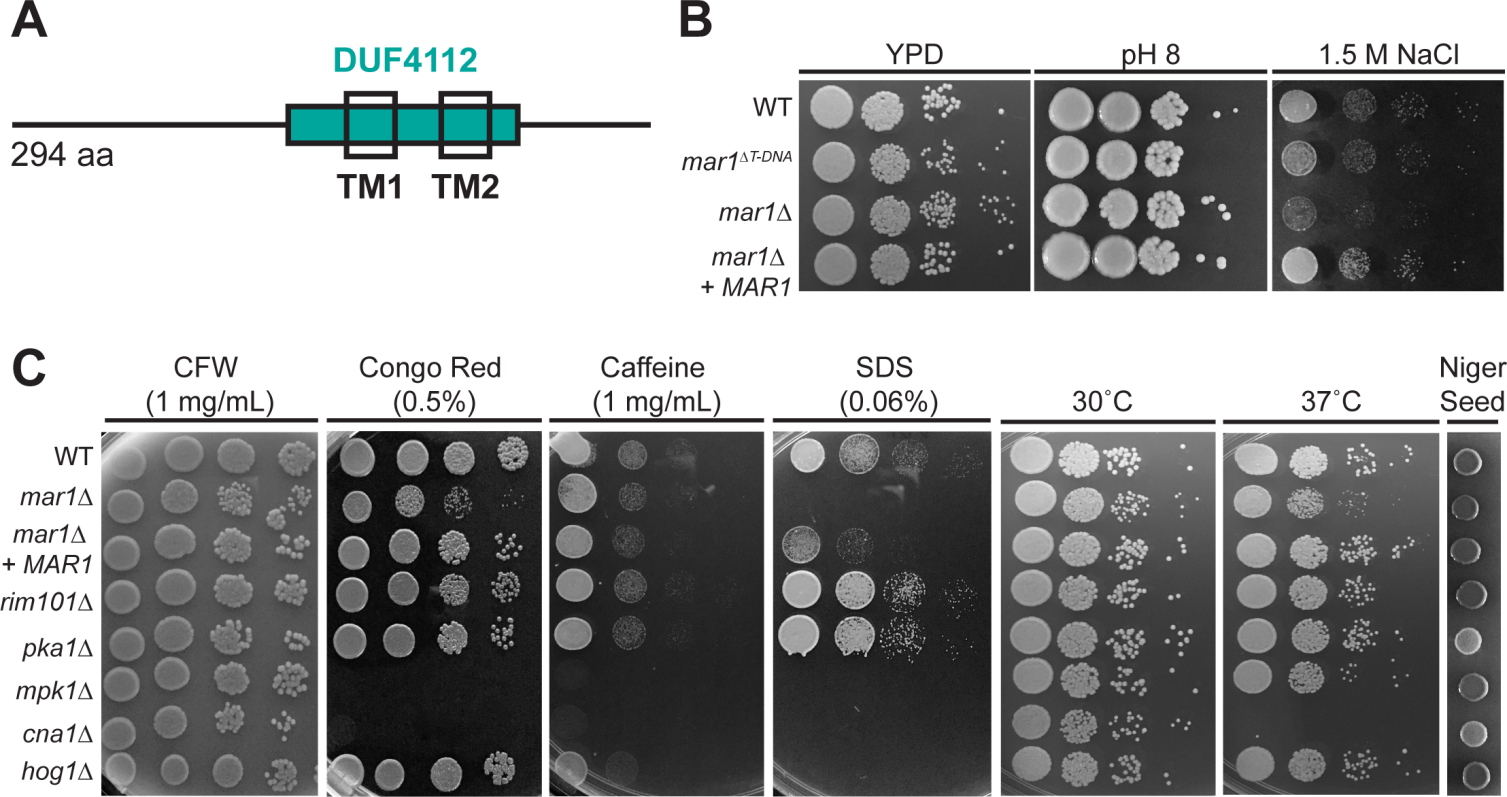
Mar1 is required for proper cell wall integrity. (A) Schematic of the Mar1 protein domains. DUF, domain of unknown function; TM, transmembrane. (B) The *mar1*^*ΔT-DNA*^ insertional mutant and a *mar1Δ* full deletion mutant are dry on pH 8 and sensitive to 1.5 M NaCl. Serial dilutions of the indicated strains were spotted onto YPD, YPD with 150 mM HEPES at pH 8, or YPD with 1.5 M NaCl. (C) The *mar1Δ* strain shares distinct and overlapping sensitivities to cell wall stressors with other cell wall integrity pathway mutants. Strains were serially diluted and spotted onto YPD with the addition of the indicated cell wall stressors or at the indicated temperatures. Melanin was assessed by the production of brown pigment on Niger Seed agar at 30°C. CFW, calcofluor white; SDS, sodium dodecyl sulfate.

From this screen, we identified a mutant displaying dry colony morphology and slight sensitivity to alkaline pH as well as decreased growth on elevated salt concentrations. This strain had a mutation in a previously uncharacterized gene (CNAG_06695), which we have named *MAR1* (macrophage activating regulator of cell wall-1). The encoded protein is predicted to contain two transmembrane domains, which comprise a larger domain of unknown function (DUF4112) (Fig 1A). However, Mar1 has no annotated predicted functions based on its sequence, and it shares no significant sequence homology with proteins from other species, including other basidiomycetes, with the exception of highly related *Cryptococcus* species (*C*. *deneoformans*, *C*. *deuterogattii*, and *C*. *gattii*). We confirmed the phenotypes of this mutant by independently disrupting the entire *MAR1* gene in the wild type background (Fig 1B). We also complemented all phenotypes by reintroduction of the wild type allele into the *mar1Δ* mutant.

We assessed the sensitivity of the *mar1Δ* mutant to common cell wall stressors. These agents included calcofluor white (CFW, binds and blocks chitin assembly), Congo red (inhibits assembly of cell wall polymers, especially chitin), caffeine (affects signal transduction and general cell wall integrity), and SDS (cell membrane stressor) [23–26]. When incubated in the presence of cell wall stressors, growth of the *mar1Δ* strain was severely inhibited by Congo red (0.5%) and SDS (0.06%) compared to the WT strain (Fig 1C). In contrast, the *mar1Δ* mutant only displayed a slight decrease in colony size on CFW (1 mg/ml), and growth was comparable to WT when incubated in the presence of caffeine (1 mg/ml).

Given the enhanced susceptibilities of the *mar1Δ* mutant to cell wall perturbing agents, we considered that the Mar1 protein might be involved in other cell-signaling pathways that regulate or respond to defects in cell wall integrity, including (1) the Rim/alkaline response pathway (2) the PKA/cAMP pathway, (3) the PKC/cell wall integrity (CWI) pathway, (4) the calcineurin pathway, and (5) the high-osmolality-glycerol (HOG) pathway. Therefore, we compared the *mar1Δ* mutant to strains with mutations in these other pathways (representative mutants used for each pathway: (1) *rim101Δ*; (2) *pka1Δ*; (3) *mpk1Δ*; (4) *cna1Δ*; (5) *hog1Δ*) to specifically test patterns of susceptibility to common cell wall stressors (Fig 1C). While sensitivity to Congo red and SDS is observed in mutants of the PKC/CWI and calcineurin pathways, these strains are also sensitive to caffeine. In a manner distinct from the SDS susceptibility of the *mar1Δ* strain, Rim and PKA/cAMP pathway mutants grew more robustly on SDS. Hog pathway strains were exclusively sensitive to SDS and no other stressors tested.

Additionally, we tested these strains for phenotypes associated with virulence including melanin production and the ability to grow at high temperature. The *mar1Δ* mutant displayed a modest growth defect when incubated at 37°C, however melanin production was comparable to WT (Fig 1C). These phenotypes contrasted sharply with calcineurin pathway mutants, which display defective thermotolerance, and PKA pathway mutants, that have defective melanin production [27,28]. Due to the modest temperature sensitivity of the *mar1Δ* strain, we also tested the effect of the Hsp90 inhibitor, radicicol, on *mar1Δ* growth at 30°C and 37°C. We observed identical MICs for WT and *mar1Δ* cells (12.5 μM at 30°C and 1.56 μM at 37°C). Together these data indicate that *MAR1* is required for normal cell wall integrity under certain cell wall stress conditions. However, this particular combination of sensitivities does not precisely mimic that of mutants in any of the previously studied cell wall responsive pathways.

### *MAR1* is required for capsule attachment

**Fig 2:**
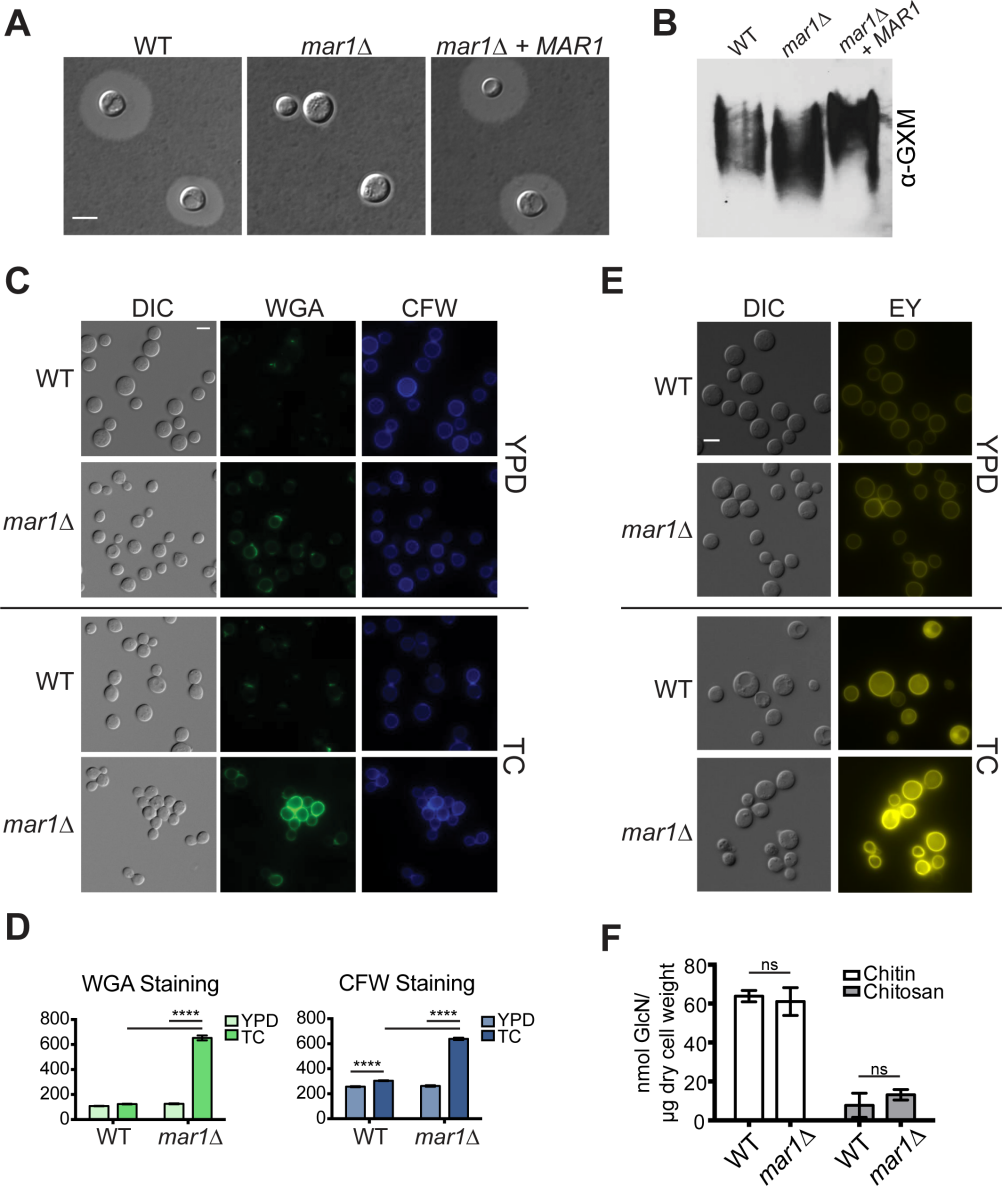
The *mar1Δ* mutant has an aberrant cell surface. (A) The *mar1Δ* strain has a capsule defect. Cells were incubated in capsule-inducing conditions (CO_2_-independent tissue culture (TC) medium, 37°C) with shaking for 72 hours. Capsule was assessed by India ink counterstaining, followed by imaging. (B) The *mar1Δ* strain sheds capsule comparable to WT. Shed capsule polysaccharide was measured by blotting of culture supernatant, using an anti-GXM antibody to probe for capsule as described previously [30]. (C) The *mar1Δ* mutant displays increased staining for exposed chitin and chitooligomers in tissue culture medium. Cells were incubated for 16–18 hours at 30°C in rich medium (YPD) or 37°C in tissue culture medium (TC), followed by staining with FITC-conjugated wheat germ agglutinin (WGA) for exposed chitin/chitooligomers, or calcofluor white (CFW) for total chitin. Stained cells were imaged by fluorescent microscopy with the appropriate filters. (D) Average fluorescence of at least 100 individual cells was measured using ImageJ/Fiji software. ****, p < 0.0001 as determined by two-way ANOVA with Tukey’s multiple comparisons test. (E) The *mar1Δ* mutant displays increased chitosan staining. Cells were incubated for 16–18 hours at 30°C (YPD) or 37°C (TC), followed by staining with eosin Y (EY) for chitosan. Stained cells were imaged by fluorescent microscopy. (F) The *mar1Δ* cell wall does not have increased total chitin or chitosan. Cells were incubated for 16–18 hours at 37°C in TC medium, followed by cell wall isolation. Chitin and chitosan levels were quantified using a modified 3-methyl-2-benzothiazolinone hydrazine hydrochloride (MBTH) colorimetric assay as described previously [16]. Data represent means of 3 independent cell wall isolations (n = 3 for each strain). Ns, not significant as determined by two-way ANOVA with Sidak’s multiple comparisons test.

The *C*. *neoformans* cell wall serves as the site of attachment for the polysaccharides that comprise the cell surface capsule [13]. Therefore, some *C*. *neoformans* strains with defects in cell wall organization and structure also display defective encapsulation. We incubated the *mar1Δ* strain in capsule-inducing tissue culture medium and assessed capsule microscopically by India ink staining. Compared to WT, the *mar1Δ* mutant displayed a marked reduction in surface capsule (Fig 2A). Capsular polysaccharide is synthesized in the cytoplasm, and then secreted, where it binds to the cell surface [29]. To differentiate between a capsule biosynthesis and capsule attachment defect, we assayed the relative amount of secreted capsule in the culture supernatant using a previously described immuno-blotting technique [21,30]. In brief, we used the mAb18B7 monoclonal antibody directed against the main capsule component GXM (glucuronoxylomannan) to probe for secreted capsule polysaccharide. By this method, we observed WT-levels of this capsular polysaccharide secreted by the *mar1Δ* mutant (Fig 2B). However, the electrophoretic mobility of this polysaccharide appears to differ slightly from the WT and complemented strains. These results indicate that *mar1Δ* has no defect in total capsule polysaccharide production, but that the defect in surface encapsulation is likely due to a defect in capsule attachment, and perhaps polysaccharide composition/structure. This pattern of altered capsule attachment is distinct from PKA pathway mutants that display impaired capsule synthesis [21]; however, capsule attachment defects have been observed in other strains with cell wall defects [13,21].

### The *mar1Δ* mutant has an aberrant cell surface

To more fully explore the cell wall changes of the *mar1Δ* mutant, we performed microscopy with stains and antibodies specific for various cell wall components. After the *mar1*Δ mutant was incubated in host-like tissue-culture medium (TC), we observed a striking increase in its staining by FITC-conjugated wheat germ agglutinin (WGA), a lectin that recognizes exposed chitin and chitooligomers (Fig 2C) [16,31]. This pattern of increased WGA staining does not occur when the cells are incubated in rich medium (YPD), indicating this chitooligomer-exposure phenotype is induced by host-like TC conditions (Fig 2C and 2D). These initial observations were quantified, demonstrating a significant increase in the average fluorescence of WGA-stained *mar1Δ* cells incubated in TC medium compared to WT cells or *mar1Δ* cells incubated in YPD (Fig 2D).

Whereas chitooligomer exposure is well approximated by quantifying cell surface binding of the large WGA molecule, total cell wall chitooligomer content is better assessed using calcofluor white (CFW), a smaller fluorescent molecule that more easily penetrates into the cell surface and binds chitin and other chitooligomers. In contrast to the marked shift in WGA staining, we only observed a slight increase in CFW staining of *mar1Δ* cells compared to WT, and only when the cells were incubated in TC medium (Fig 2C and 2D).

We also measured levels of chitosan, the de-acetylated form of chitin, by Eosin Y (EY) staining (Fig 2E) [32]. Similar to staining for chitin, we did not observe a major change in fluorescence when cells were incubated in YPD (Fig 2E); however, we observed enhanced staining of *mar1Δ* cells incubated in TC medium compared to WT cells. Together these cell wall staining results show that in host-like tissue culture conditions the *mar1Δ* strain has increased exposure of chitooligomers, including both chitin and chitosan.

### The *mar1Δ* mutant cell wall changes in tissue culture medium are induced by pH and glucose deprivation

**Fig 3:**
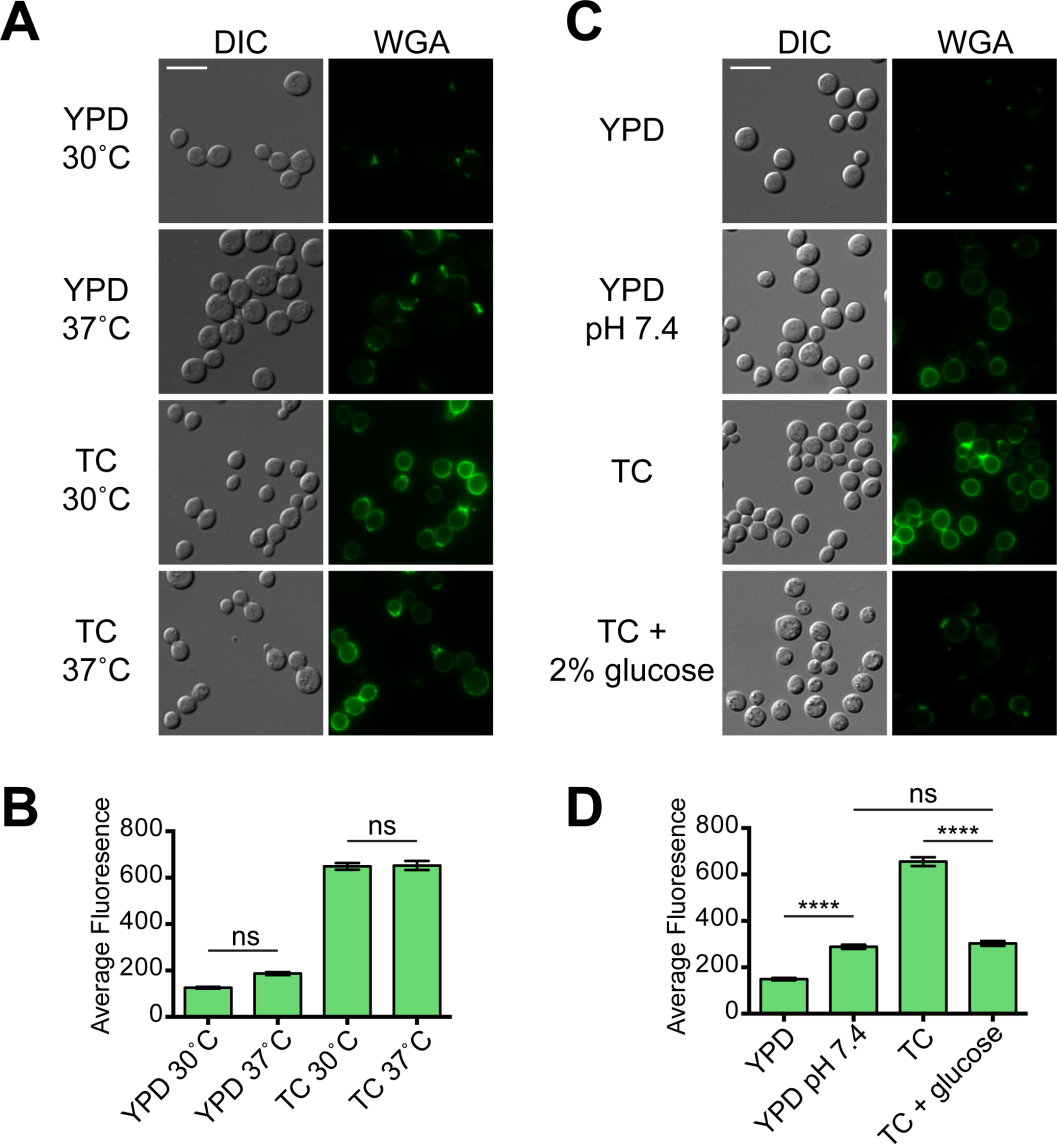
Cell wall changes in the *mar1Δ* mutant induced in tissue culture medium are dependent on pH and glucose deprivation. (A) The *mar1Δ* cell wall changes are not dependent on temperature. The *mar1Δ* strain was incubated for 16–18 hours with shaking at the indicated temperature in rich medium (YPD) or tissue culture medium (TC). Exposed chitooligomers in the cell wall were stained with FITC-conjugated WGA and imaged by fluorescent microscopy using the GFP filter. (B) Average fluorescence of at least 100 individual cells was measured using ImageJ/Fiji software. Ns, not significant as determined by one-way ANOVA with Tukey’s multiple comparisons test; all other comparisons, p < 0.0001. (C) The *mar1Δ* cell wall changes occur with increased pH and can be partially suppressed by glucose supplementation. *mar1Δ* cells were incubated for 16–18 hours with shaking at 30°C in YPD, YPD buffered to pH 7.4, TC, or TC supplemented with 2% glucose. Cells were stained with FITC-conjugated WGA and imaged as above. Bar, 10 μM. (D) Average fluorescence was measured as above. ****, p < 0.0001; ns, not significant, as determined by one-way ANOVA with Tukey’s multiple comparisons test.

To better understand how host-like tissue culture conditions induce cell wall changes in the *mar1Δ* mutant, we sought to better define what component of this condition is responsible. To determine if the *mar1Δ* cell wall changes were dependent on temperature, we incubated cells in YPD or TC media at 30°C and 37°C, and measured staining of exposed chitooligomers by WGA (Fig 3A and 3B, S1A Fig). We observed a small, but not significant, increase in staining of cells incubated in YPD at 37°C. By contrast, cells incubated in TC regardless of temperature displayed significant and equivalent increases in staining. These data indicate that increased temperature is not the major driver of the *mar1Δ* cell wall changes.

We next tested the role for pH in inducing cell wall changes. Standard YPD medium has a pH of 5–6, while TC medium is buffered to pH 7.4. To test if increased pH could induce the *mar1Δ* mutant cell wall changes, we buffered YPD to pH 7.4 and measured staining by WGA (Fig 3C and 3D, S1A Fig). We observed a small, but significant increase in chitooligomer staining of *mar1Δ* cells, suggesting that increased pH is involved in TC-induced cell wall changes. Interestingly, when we buffered YPD to pH 8, we observed increased WGA staining of both WT and *mar1Δ* cells (S1B Fig).

While higher pH induced increased WGA staining, it did not fully recapitulate the level of staining observed in TC medium. We next tried to suppress the *mar1Δ* cell wall changes by supplementing TC medium with common nutrients found in rich medium. We observed that when we incubated *mar1Δ* cells in TC medium supplemented with 2% glucose, the intensity of WGA staining was partially suppressed (Fig 3C and 3D). We did not observe any changes in WT staining or any suppression in TC medium supplemented with 1x complete amino acids (S1A and S1B Fig). Together these data indicate that a combination of increased pH and decreased glucose availability in TC medium induces increased chitooligomer exposure of the *mar1Δ* mutant.

### The *mar1Δ* cell wall has decreased levels of α- and β-glucan, but not chitin or chitosan

We used several independent methods to further quantify the relative levels of cell wall carbohydrates in our strains. First, we used an enzymatic method [17] to quantify total chitin and chitosan levels in the *mar1Δ* and WT cell walls after incubation in TC medium, the condition in which we observe differences in staining intensity. Compared to the WT, the *mar1Δ* mutant displayed no increase in total chitin or chitosan using this biochemical assay (Fig 2F).

**Fig 4:**
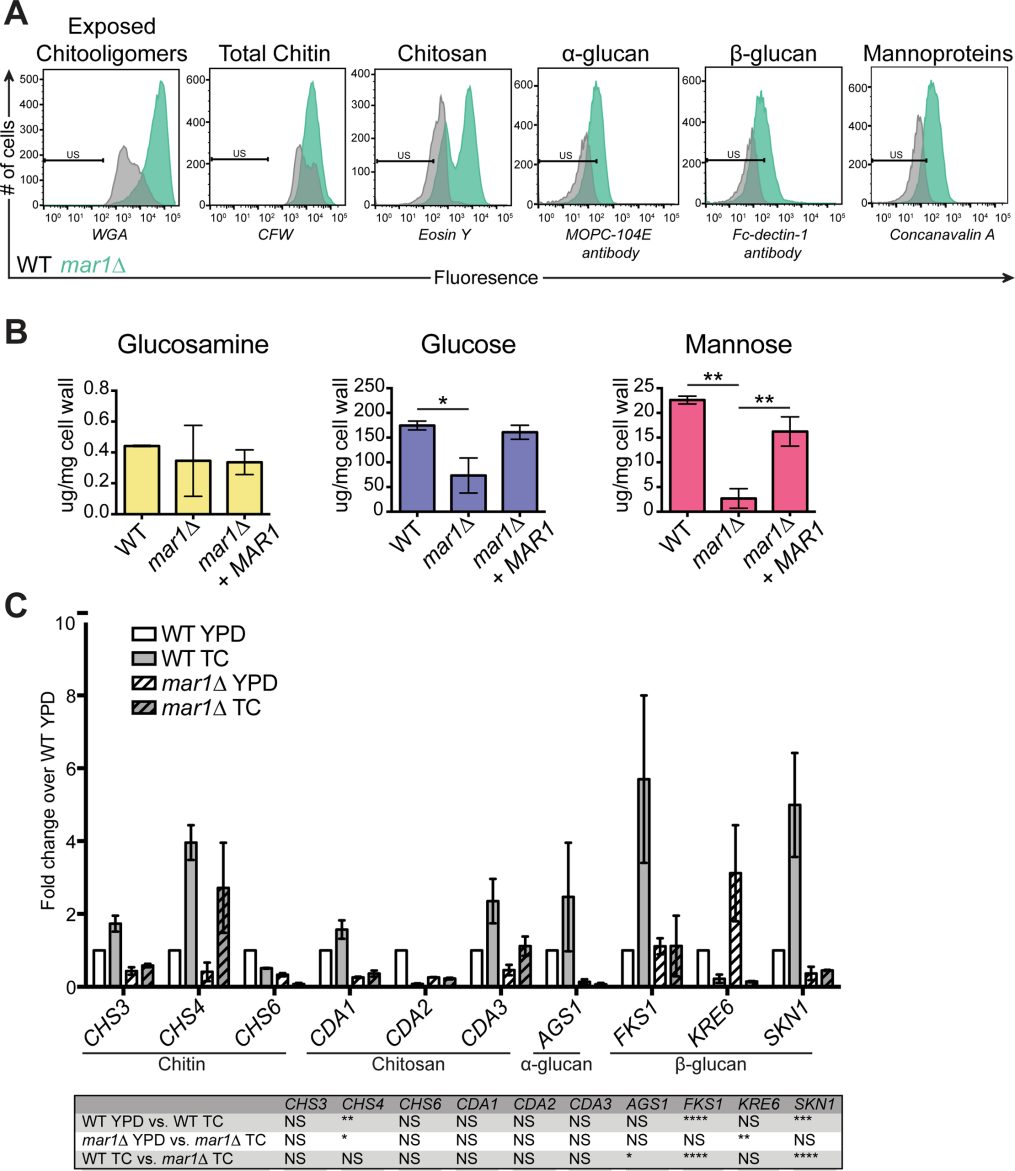
Cell wall components are altered in the *mar1Δ* cell wall. (A) The *mar1Δ* cell wall has increased chitin and chitosan staining by flow cytometry, but *mar1Δ* staining for α-glucans, β-(1,3)-glucan, and mannoproteins is limited above baseline. WT and *mar1Δ* cultures were incubated for 16–18 hours at 37°C in TC medium, fixed, labeled, and analyzed by flow cytometry. WGA was used to stain exposed chitin and CFW was used to stain total chitin. EY was used to stain chitosan. An MOPC-104E antibody with an anti-mouse AlexaFluor 488 secondary antibody was used to label α-glucan. An Fc-Dectin-1 fusion protein coupled with an anti-human AlexaFluor 488 secondary antibody was used to label β-(1,3)-glucan. Concanavalin A conjugated to AlexaFluor 488 was used to label mannoproteins. Relevant events were gated in the FSC/SSC plots and are represented as histograms with mean fluorescence on the x-axis and cell counts on the y-axis. Unstained cells were sorted as controls to determined positive labeling. (B) The *mar1Δ* cell wall has decreased glucan and mannan. Cells were incubated for 16–18 hours at 37°C in TC medium, followed by cell wall isolation. Cell wall carbohydrate levels were quantified using high performance anion-exchange chromatography with pulse ampherometric detection (HPAEC-PAD). All data represent means of results from 3 independent cell wall preparations for each strain. *, p < 0.05; **, p < 0.01 as determined by one-way ANOVA with Tukey’s multiple comparisons test. All other comparisons, not significant (C) Cell wall genes are differentially regulated in *mar1Δ*. A concentration of 10^7^ cells/ml in 25 ml YPD (30°C) or TC (37°C) were incubated for 1.5 hours, followed by RNA extraction and cDNA synthesis. Expression of cell wall biosynthesis genes was determined by real-time PCR. Fold change was calculated relative to WT YPD levels and normalized to the expression of an internal control. Data represent means of results from 2 independent *C*. *neoformans* cultures and RNA extractions per strain. *, p < 0.05; **, p < 0.01; ***, p < 0.001; ****, p < 0.0001 as determined by two-way ANOVA with Tukey’s multiple comparisons test.

We also quantified our initial staining observations using flow cytometry, demonstrating a striking increase in the mean fluorescence intensity (MFI) of WGA-stained *mar1Δ* cells compared to WT (Fig 4A). Similar to what we saw by staining, we observed a modest increase in the MFI of CFW-stained *mar1Δ* cells (Fig 4A). Interestingly, we observed two peaks of EY-stained *mar1Δ* cells, one corresponding to the mean fluorescence intensity (MFI) of WT stained cells, and a second shifted to a higher MFI (Fig 4A). This suggests a non-homogeneous pattern of chitosan exposure after incubation in host-like conditions in *mar1Δ* cells. We were unable to accurately assess the relative levels of other cell wall carbohydrates such as α-glucan, β-glucan, and mannoproteins using staining and microscopy methods adapted from other fungal species. In all cases, the staining signal of WT cells was similar to unstained controls (Fig 4A). Therefore, we utilized biochemical methods to quantify cell wall carbohydrate composition. We extracted total cell wall material from our strains and used high-performance liquid chromatography (HPLC) to quantify the levels of glucosamine (chitin and chitosan together), glucose (glucans), and mannose (mannosylated proteins) in these extracts [16,33]. Similar to our enzymatic measurements of chitin and chitosan, we observed no significant difference in the level of glucosamine between the WT and *mar1Δ* strains (Fig 4B). However, by HPLC, we measured a significant decrease in the levels of glucose and mannose in the *mar1Δ* mutant compared to the WT (Fig 4B). Together, these results suggest that Mar1 is required to maintain normal levels of the outer glucan and mannan cell wall layers. Accordingly, in the absence of functional Mar1, the resulting changes in outer cell wall carbohydrate abundance result in increased exposure, but not total levels, of the inner cell wall chitooligomers, chitin and chitosan.

### Mar1 regulates α- and β-glucan synthase expression

To determine the site of regulation for these cell wall changes, we performed quantitative real time PCR analysis of selected cell wall synthesis genes in the WT and *mar1Δ* strains. Interestingly, as the WT strain transitions from a rich medium to tissue culture medium, there is a marked increase in the expression of genes encoding α- and β-glucans (Fig 4C). In contrast, there is no significant transcriptional change in most chitin synthase and chitin deacetylase genes during this environmental transition, except for the *CHS4* chitin synthase. Consistent with our chitooligomer cell wall staining and quantification data, the expression of these chitin synthase and chitin deacetylase genes was not significantly different between WT and *mar1Δ* in tissue culture medium. By contrast, the genes encoding the major glucan synthases, including the α-glucan synthase *AGS1* and the β-1,3-glucan synthase *FKS1*, displayed statistically significantly decreased expression in tissue culture medium in *mar1Δ* compared to WT. These data indicate that Mar1 is required for the transcriptional induction of glucan cell wall genes, including *AGS1* and *FKS1*, that are typically upregulated during host-like conditions.

### Rim and cell wall integrity pathway signaling is intact in the *mar1Δ* background

We chose to more fully explore the relationship of Mar1 function with the Rim and CWI signaling pathways given our observation that the mutant strains associated with each of these pathways most closely shared cell wall-related phenotypes with the *mar1Δ* strain. *C*. *neoformans* strains defective in Rim pathway signaling are unable to grow well at alkaline pH or in the presence of elevated salt concentrations, similar to the *mar1Δ* strain. This signaling pathway is responsible for activating the Rim101 transcription factor, which in turn regulates the expression of many genes required for cell wall integrity and organization during growth in host-like conditions. Additionally, Rim pathway mutants have increased chitooligomer exposure [16].

The *MAR1* gene was not identified as a Rim101 target in previously published comparative transcriptional profiling experiments [14]. However, direct analysis of the raw data from these experiments (NCBI GEO database accession number GSE43189) indicated that the *MAR1* locus was not included in the statistical analyses due to its relatively low transcript abundance. To more definitively determine whether *MAR1* is a downstream target of the Rim101 transcription factor, we first performed quantitative real time PCR to measure the expression of *MAR1* in the WT background in YPD and TC media. We observed that *MAR1* had significantly induced expression in TC medium compared to YPD (p = 0.0017) (S2A Fig). To next determine if the expression of *MAR1* is regulated in a Rim101-dependent manner, we analyzed updated transcriptional profiling of the *rim101Δ* strain in TC medium recently carried out in our laboratory (NCBI GEO database accession number GSE110723). Expression of the *MAR1* transcript in the *rim101Δ* mutant versus WT was only modestly reduced (log_2_-fold change = 0.772, p = 0.04), suggesting that *MAR1* is likely not a major target of Rim101.

We also assessed whether Mar1 might function in the Rim signaling pathway upstream of Rim101. Rim pathway activation occurs in a pH-dependent manner, culminating in the cleavage of the Rim101 transcription factor and its translocation to the nucleus [34]. Rim101 cleavage and nuclear localization are disrupted in mutants of upstream Rim pathway activators [35]. In contrast, Rim101 cleavage/activation is intact in the *mar1Δ* background (S2B Fig). Together these data indicate that Mar1 likely operates in cellular processes independent of the *C*. *neoformans* Rim pathway, despite phenotypic similarities of these mutant strains.

The PKC/CWI pathway is responsible for the activation of the Mpk1 MAP kinase protein, which in turn coordinates enhanced cell wall stress resistance. Accordingly, mutants in this pathway display defective phosphorylation of Mpk1. We therefore assessed Mpk1 phosphorylation in response to cell wall stress in the WT and *mar1Δ* mutant strains. Western blots of total cell lysates from these strains were analyzed using an antibody directed against phosphorylated (activated) Mpk1 [36,37]. After 3.5 hours of incubation in YPD, WT and *mar1Δ* cells had a modest level of phosphorylated Mpk1 (S3A Fig). After incubation in TC medium, both WT and *mar1Δ* displayed enhanced Mpk1 phosphorylation (S3A Fig). These results demonstrate that Mar1 is not required for CWI integrity pathway activation under host-like tissue culture conditions as measured by Mpk1 phosphorylation. To further test this, we constructed a *mar1Δ mpk1Δ* double mutant and analyzed chitooligomer exposure by WGA staining. In agreement with the *mar1Δ* mutation inducing distinct cell wall changes, the *mar1Δ mpk1Δ* double mutant displayed more WGA staining then either *mpk1Δ* or *mar1Δ* single mutants (S3B Fig). The patterns of Rim and CWI signaling pathway activation in the *mar1Δ* strain suggest that the Mar1 protein regulates cell wall integrity in a novel manner.

### Mar1 localizes to intracellular and cell surface puncta

**Fig 5:**
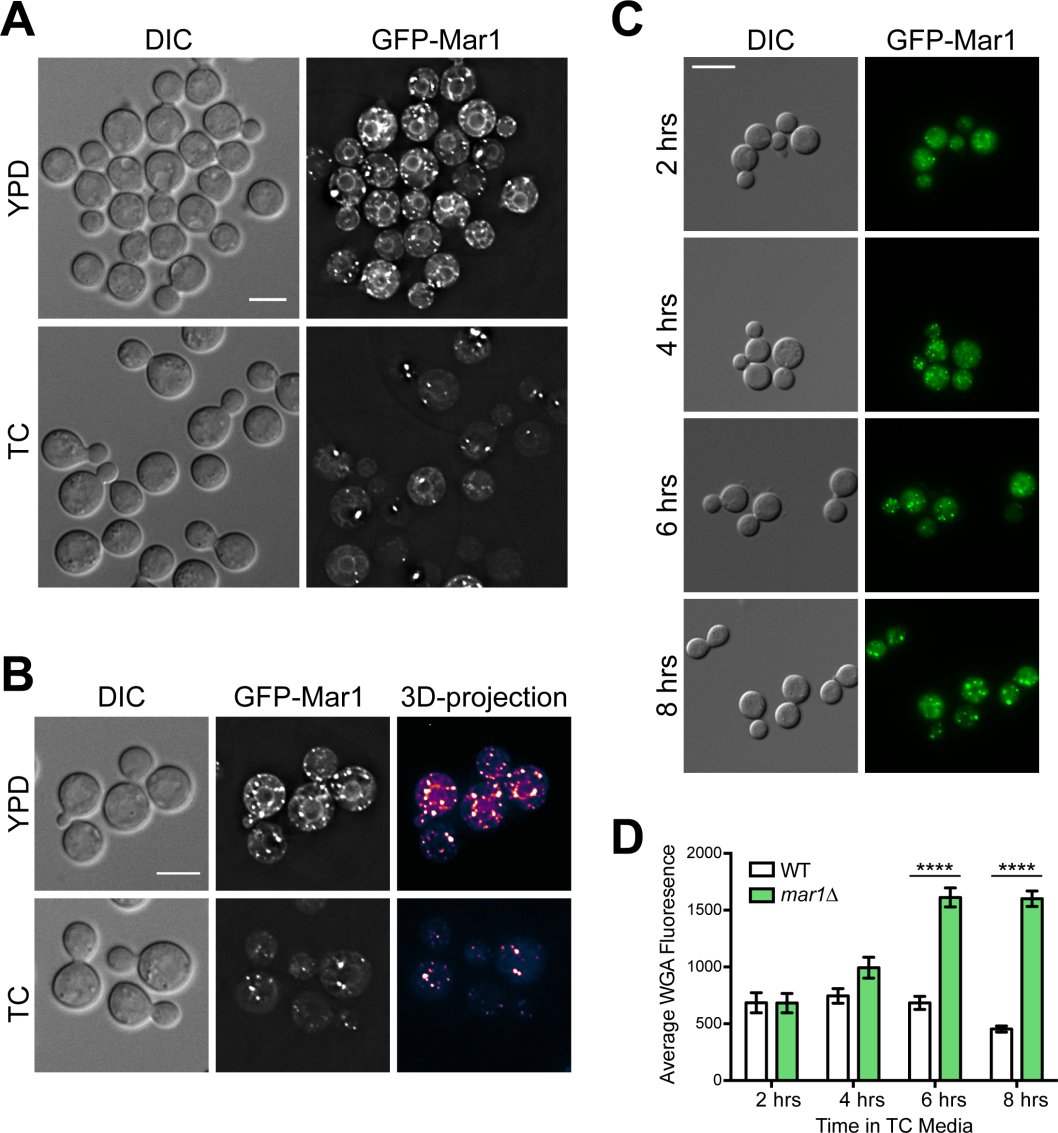
Mar1 protein localization is dynamic over time in tissue culture medium. (A) Localization of a Mar1-GFP fusion protein was assessed after cells were incubated for 16–18 hours in YPD (30°C) or TC (37°C). Live cells were imaged using DeltaVision deconvolution fluorescent microscopy with the GFP filter. Images were deconvolved using softWoRx software. Bar, 10 μM. (B) Cells were incubated and imaged as above. 3D-projections were generated from z-stacked images using ImageJ/Fiji software and are pseudo-colored to indicate brightness of staining from yellow to blue. Bar, 10 μM. (C) A Mar1-GFP expression strain was pre-incubated in YPD medium to mid-log phase and transferred to TC medium at time zero. Cells were incubated for the indicated time in TC at 37°C with shaking and imaged by fluorescent microscopy using the GFP filter. Bar, 10 μM. (D) Quantification of staining by FITC-conjugated WGA. The WT and *mar1Δ* strains were incubated to mid-log phase in YPD medium and transferred to TC medium at time zero. Exposed chitooligomers in the cell wall were stained with FITC-conjugated WGA, imaged by fluorescent microscopy using the GFP filter, and average fluorescence was quantified by ImageJ/Fiji software. ****, p < 0.0001 as determined by two-way ANOVA with Sidak’s multiple comparisons test.

To better understand the function of Mar1, we generated an N-terminally tagged GFP-Mar1 fusion protein and analyzed its localization by microscopy. We expressed this fusion protein under the constitutively active *HIS3* promoter in the *mar1Δ* mutant strain background. This fusion protein was functional, displaying partial suppression of *mar1Δ* dry colony morphology on pH 8 plates. After overnight incubation in YPD medium, GFP-Mar1 localized throughout the cell to small punctate structures on endomembranes and at the cell surface (Fig 5A). 3D-projection of Z-stacked images indicated that many of the observed puncta were located on the cell surface (Fig 5B). Compared to what was observed in YPD-incubated cells, the number of GFP-Mar1 puncta was decreased after incubation in TC medium (Fig 5A and 5B). These puncta also appeared larger and more globular in nature, and overall intracellular endomembrane staining was less intense after incubation in TC medium.

To further characterize the dynamics of GFP-Mar1 protein localization, we analyzed protein fluorescence over time after shifting cells to TC medium (Fig 5C). After 2 and 4 hours in TC medium, GFP-Mar1 localizes to small punctate structures as well as to endomembranes, similar to what we observed for cells incubated in YPD overnight. After 6 hours, some GFP-Mar1 puncta begin to appear globular, more similar to what we observed for cells incubated in TC overnight. By 8 hours, the majority of GFP-Mar1 puncta appear globular. Interestingly, when we measured *mar1Δ* chitooligomer exposure over time in TC medium, we observed a similar time course of changes (Fig 5D). Increased WGA staining of *mar1Δ* cells can be observed beginning around 6 hours after incubation in TC medium, similar to when globular GFP-Mar1 puncta are seen.

### Intracellular trafficking is impaired in *mar1Δ*

**Fig 6:**
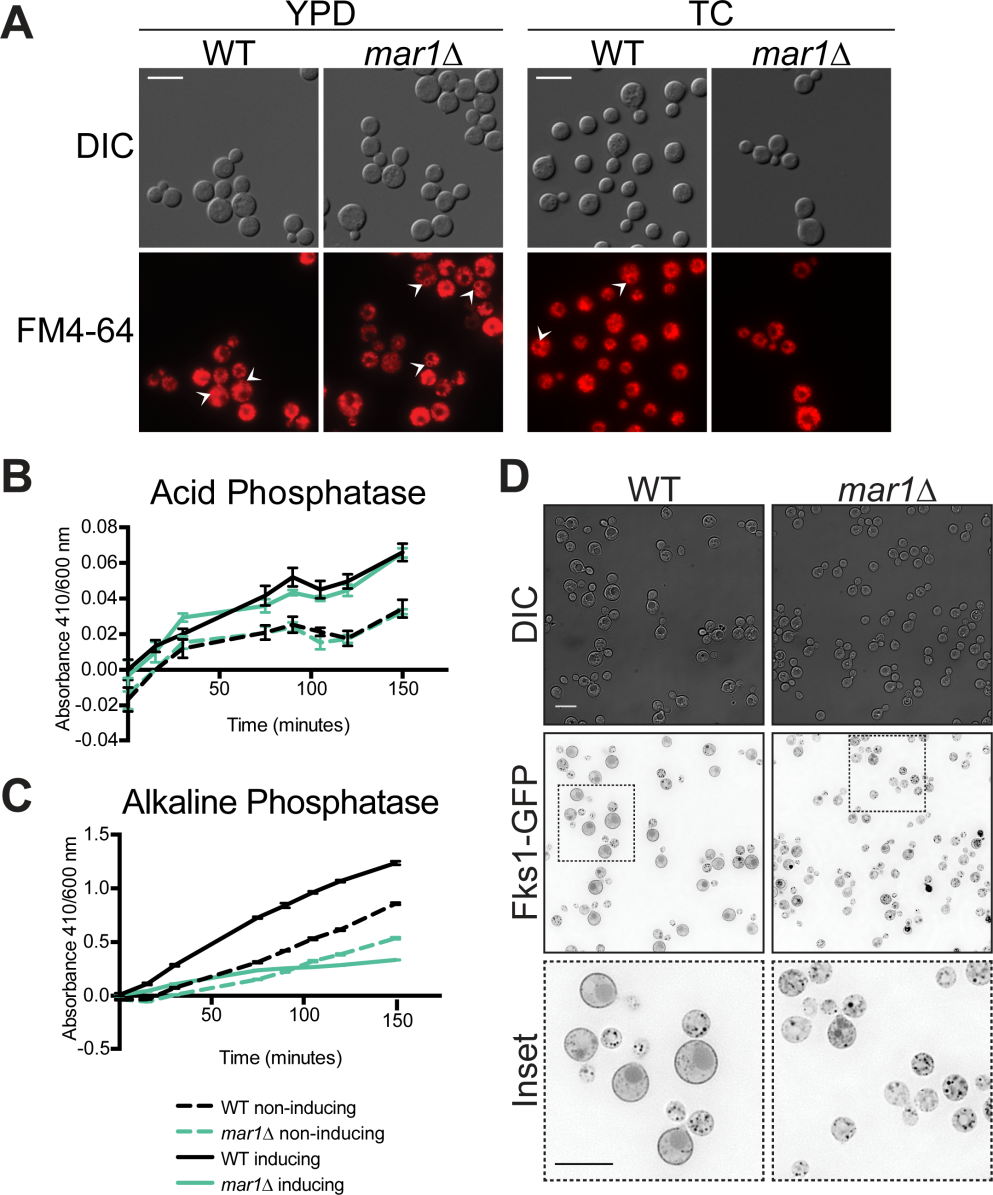
Intracellular trafficking and localization of the β-(1,3)-glucan synthase, Fks1, are impaired in the *mar1Δ* mutant. (A) Uptake of the lipophilic dye, FM4-64, is irregular in *mar1Δ* cells. Cells were incubated overnight in YPD (30°C) or TC (37°) and stained with FM4-64 for 30 minutes with shaking, followed by pelleting and refreshing in the indicated media for an additional 30 minutes with shaking. Stained cells were imaged by fluorescent microscopy with the Texas Red filter and were taken with the same exposure time. Bar, 10 μM. Arrows indicate endocytic vesicles (B, C) Acid phosphatase secretion is intact in the *mar1Δ* strain; alkaline phosphatase activity is decreased. WT and *mar1Δ* cultures were pre-incubated for 16–18 hours in phosphate replete minimal medium. The cells were diluted to an OD of 0.9 in either phosphate replete (non-inducing) or phosphate deficient (inducing) minimal medium and incubated for 3 hours at 30°C with shaking. (B) Acidic or (C) alkaline *para*-Nitrophenylphosphate (pNPP) substrate solution was added to each well and plates were incubated for an additional 2.5 hours at 37°C with shaking. Phosphatase activity was measured as absorbance at 410 nm over cell density at 600 nm. Data represent the mean of 3 replicates per strain per condition. (D) Fks1 is mislocalized in TC medium in *mar1Δ* cells. The Fks1-GFP fusion protein was expressed in the WT and *mar1Δ* mutant strains. Cells were incubated for 16–18 hours in TC medium at 37°C. Live cells were imaged using DeltaVision deconvolution fluorescent microscopy with the GFP filter. Images were deconvolved using softWoRx software. Bar, 10 μM.

The dynamic localization of GFP-Mar1 led us to next investigate intracellular trafficking processes in the *mar1Δ* mutant strain. To determine if these processes were impaired, we used the lipophilic dye FM4-64 to assess rates of endocytosis in YPD and TC media (Fig 6A). Medium-sized, bright endocytic vesicles can be seen after 30 minutes of staining in WT and *mar1Δ* cells incubated in YPD. Endocytic vesicles can also be observed in WT cells incubated in TC medium; However, by contrast, less well-defined, tubular structures are observed in *mar1*Δ cells incubated in TC medium.

To further assess intracellular trafficking, we measured acid and alkaline phosphatase activity in cell supernatants. In *S*. *cerevisiae*, acid phosphatase is secreted through the canonical secretory pathway, whereas alkaline phosphatase is trafficked to the vacuolar membrane through the alternative ALP pathway [38]. Both enzymes are induced under low phosphate conditions and can be assayed by measuring the colorimetric hydrolysis of the *para*-Nitrophenylphosphate (pNPP) substrate. Over a time-course of incubation with the pNPP substrate in acidic medium, phosphate starved (induced) WT and *mar1Δ* cells showed increased phosphatase activity compared to phosphate replete (non-induced) cells (Fig 6B). A similar induced increase in phosphatase activity was observed for WT cells in alkaline conditions; however, there was a minimal difference in phosphatase activity between phosphate starved and phosphate replete *mar1Δ* cells in alkaline conditions, suggesting a defect in secreted alkaline phosphatase activity (Fig 6C). Together, these data are consistent with defects in endocytic and vesicular trafficking in the *mar1Δ* mutant, but not a defect in classical secretion.

### Localization of the Fks1 β-(1,3)-glucan synthase is altered in *mar1Δ*

To further explore the *mar1Δ* mutant defect in glucan abundance, we constructed a C-terminally tagged Fks1-GFP fusion protein. We transformed this allele into the WT H99 background and selected those transformants in which the allele replaced the endogenous locus for further analysis. Previous work has indicated that *FKS1* is an essential gene in *C*. *neoformans* [39]. Therefore, the ability to replace the WT *FKS1* allele with the *FKS1-GFP* allele suggests that this fluorescent fusion protein is functional. We subsequently mutated the *MAR1* gene in the strain expressing Fks1-GFP, and we analyzed the localization of Fks1-GFP in two independent *mar1Δ* mutants. After incubation in YPD medium, we observed by fluorescent microscopy that Fks1-GFP localizes to punctate structures on cellular membranes in both the WT and *mar1Δ* backgrounds (S4A Fig). After incubation in TC medium, Fks1-GFP localization is more heterogeneous among the cell population. It is still present in puncta throughout the cell in the majority of cells, but it is enriched at the plasma membrane of some cells in the WT background, predominately mother cells (Fig 6D). By contrast, Fks1-GFP localization is more homogeneous among cells in the *mar1Δ* background, with reduced plasma membrane puncta and very few cells displaying the uniform plasma membrane localization observed in the WT (Fig 6D and S4B Fig). These data suggest that Mar1 is required for proper trafficking and localization of Fks1 to the cell membrane in TC medium.

### Cell wall ultrastructure and vesicular trafficking appear disordered in *mar1Δ* cells

**Fig 7:**
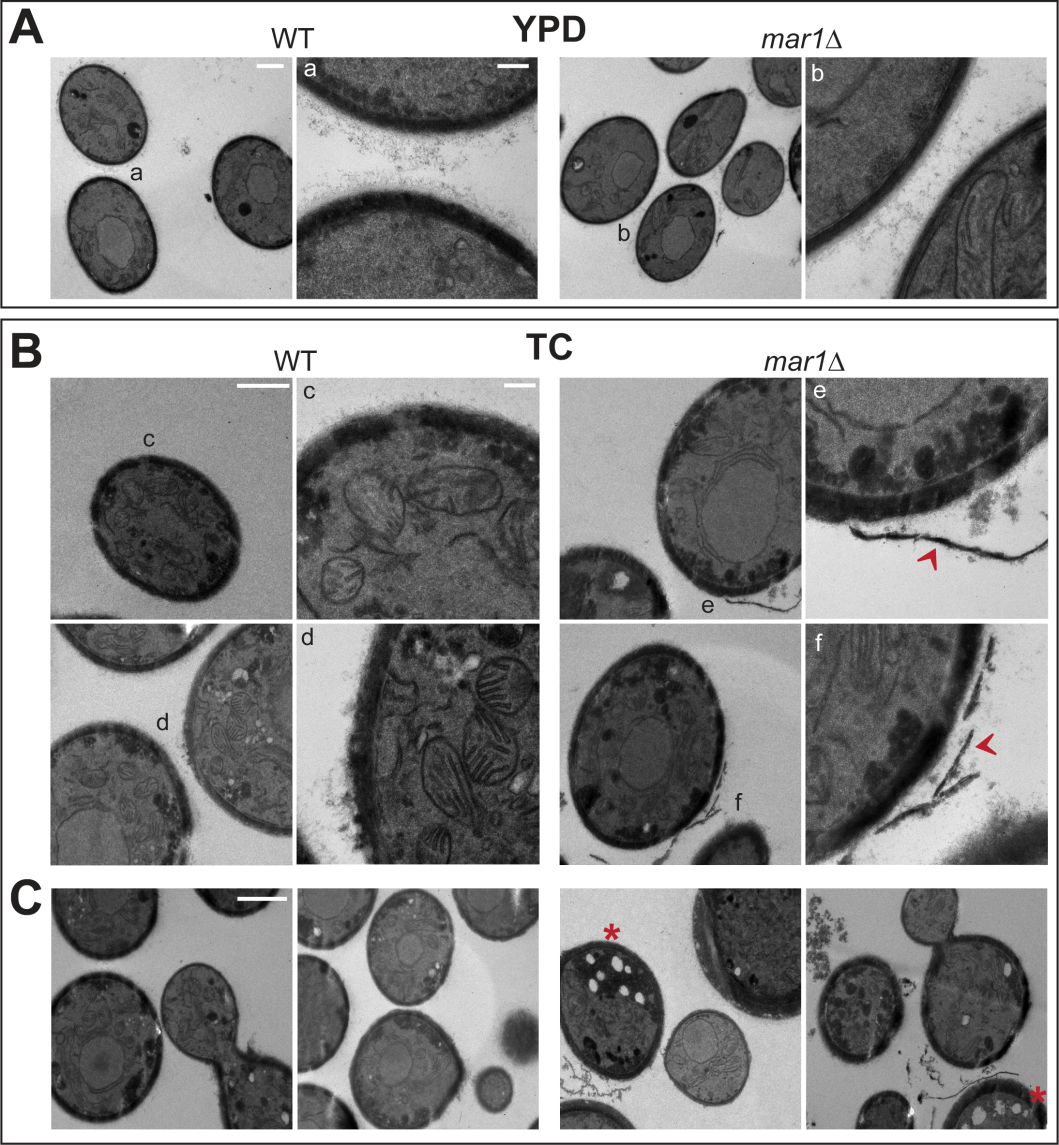
*mar1Δ* cells have disordered cell walls and altered vesicular trafficking by ultrastructure analysis. WT and *mar1Δ* cells were incubated for 16–18 hours in YPD (30°C) and TC (37°C) medium, followed by glutaraldehyde and KMnO_4_ fixation and partial dehydration as described previously [14,68,101]. Samples were further processed, embedded, sliced, and imaged by transmission electron microscopy (TEM). (A) YPD incubated cells display thin, ordered cell walls. Left image of each pair: bar, 1 μM. Right image of each pair: bar, 200 nm. Letters indicate location of inset. (B) *mar1Δ* cells incubated in TC medium have a less organized cell surface. Left image of each pair: bar, 1 μM. Right image of each pair: bar, 200 nm. Letters indicate location of inset. Red arrows indicate apparent cell wall material disassociating from cell surface. (C) There are increased numbers of electron lucent structures in *mar1Δ* cells incubated in TC medium. Bar, 1 μM. Red asterisks indicate *mar1Δ* cells with several electron lucent vesicles near cell periphery.

To better visualize the cell wall changes of the *mar1Δ* mutant, we examined our strains by transmission electron microscopy (TEM) after incubation in either YPD or TC media. In rich YPD medium, both WT and *mar1Δ* cells had thin, organized cell walls with a well-defined lamellar appearance (Fig 7A). The *mar1Δ* cell walls displayed a trend to be slightly thinner than WT cell walls in this condition, but this difference was not statistically significant (p = 0.3431). In TC medium, the cell walls of WT cells remained compact and well-ordered (Fig 7B). In contrast, *mar1Δ* cell walls were less compact and well-organized after incubation in TC medium and significantly thicker than WT or *mar1Δ* cell walls incubated in YPD (p = 0.0011 and 0.0007 respectively). Several cells appeared to have layers of cell wall material sloughing away from the cell periphery, a phenotype that was not observed for WT cells (Fig 7B). Accordingly, a significant amount of debris was also observed in the space between cells in the *mar1Δ* TC samples, likely representing degraded cell wall material, or perhaps changes in chemical cross-linking resulting in altered cell wall integrity during sample processing.

In addition to the notable TC-induced cell wall changes in the *mar1*Δ strain, the TEM images also suggested alterations in vesicular trafficking. In both WT and *mar1*Δ strains, there were numerous membrane-bound vesicles. These structures have been suggested to be secretory vesicles transporting cargo toward the cell surface [40]. In TC medium, both the WT and *mar1Δ* strains demonstrated a relative increase in the number of these vesicles carrying electron-dense material localized near the cell surface. In *mar1Δ* cells, an increase in large electron-lucent vesicles was also observed (Fig 7C). In some cells, multiple large “empty” vesicles can be seen near the cell periphery. In *S*. *cerevisiae*, the accumulation of similar vesicles has been observed for several secretory mutants [41,42]. Similarly, the *sav1Δ* secretory mutant in *C*. *neoformans* accumulates post-Golgi secondary vesicles [43]. Along with the altered FM4-64 staining and alkaline phosphatase secretion described, these images support a defect in intracellular trafficking in this mutant.

### The *mar1Δ* mutant cell wall changes are associated with increased recognition by macrophages and dendritic cells

**Fig 8:**
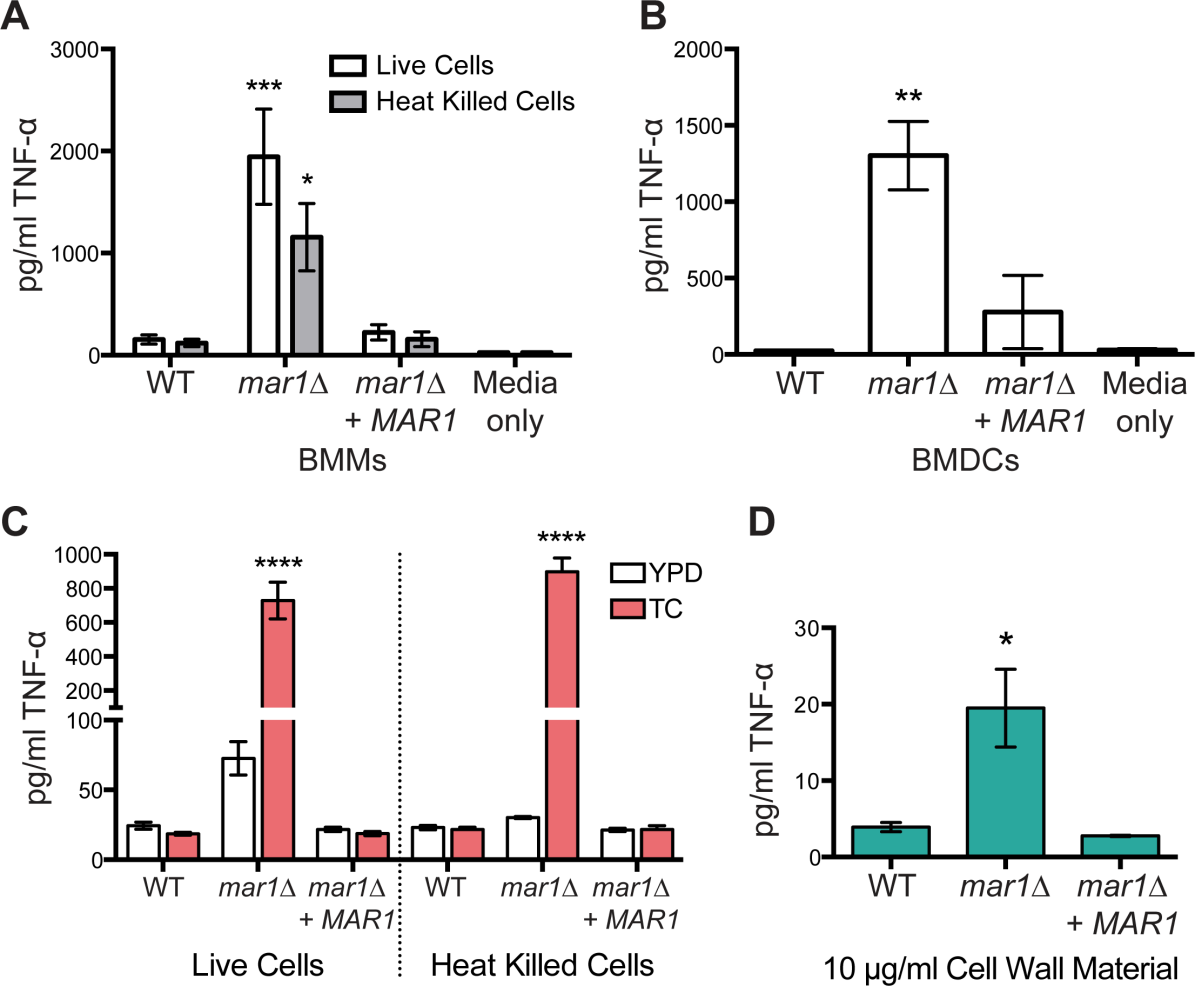
The *mar1Δ* mutant induces increased TNF-α production from macrophages and dendritic cells. (A) Macrophage activation by *mar1Δ* cells is not dependent on cell viability. WT, *mar1Δ*, and *mar1Δ + MAR1* cells were incubated for 16–18 hours at 37°C in TC medium. Bone marrow-derived macrophages (BMMs) were co-incubated with live or heat-killed (HK, 1–2 hours at 65°C) *C*. *neoformans* strains at a multiplicity of infection (MOI) of 10:1, *C*. *neoformans*:BMMs. TNF-α levels (pg/ml) were assayed from the supernatant by ELISA. Data represent means from 3 replicates per strain per condition from 3 independent experiments (n = 9). *, p < 0.05; ***, p < 0.001 *mar1Δ* vs. WT as determined by two-way ANOVA with Tukey’s multiple comparisons test. (B) *mar1Δ* induces increased TNF-α production by dendritic cells. *C*. *neoformans* cells were incubated as described above and co-incubated with bone marrow derived dendritic cells (BMDCs) at an MOI of 10:1, *C*. *neoformans*:BMDCs. TNF-α levels were assayed from the supernatant by ELISA. Data represent means from 3 replicates per strain from 2 independent experiments (n = 6). **, p < 0.01 *mar1Δ* vs. WT as determined by one-way ANOVA with Tukey’s multiple comparisons test. (C) Macrophage activation by *mar1Δ* cells is dependent on pre-culturing in TC medium. Cells were incubated for 16–18 hours in YPD (30°C) or TC (37°C) prior to co-incubation with BMMs and TNF-α quantification by ELISA as above. Data represent means from 3 replicates per strain (n = 3). ****, p < 0.0001 *mar1Δ*/TC vs. WT/ TC as determined by two-way ANOVA with Tukey’s multiple comparisons test. *mar1Δ*/YPD vs. WT/YPD, not significant. (D) Cell wall material isolated from *mar1Δ* induces increased macrophage activation. WT, *mar1Δ*, and *mar1Δ + MAR1* reconstituted cells were incubated for 16–18 hours at 37°C in TC medium, followed by cell wall isolation. 10 μg/ml of cell wall material was co-cultured with BMMs and TNF-α was quantified from the supernatant by ELISA. Data represent means of 3 independent cell wall isolations in 3 independent experiments (n = 9 for each strain). *, p < 0.05 *mar1Δ* vs. WT as determined by one-way ANOVA with Tukey’s multiple comparisons test.

Macrophages and dendritic cells are likely the first immune cells that *C*. *neoformans* contacts within infected lungs. To determine if the changes in the *mar1Δ* cell wall would affect this interaction, we quantified the production of tumor necrosis factor-alpha (TNF-α) after co-culturing WT and *mar1Δ* strains with primary bone marrow-derived macrophages (BMMs) and bone marrow-derived dendritic cells (BMDCs). We have previously used this pro-inflammatory cytokine as a marker of macrophage activation *in vitro*, observing that *C*. *neoformans* strains with increased chitooligomer exposure often induce more TNF-α production than WT [14,16]. Accordingly, we observed that *mar1Δ* cells induced significantly more TNF-α production from both BMMs and BMDCs, compared to the WT or reconstituted strains (Fig 8A and 8B). To determine if this is due to an active cellular process, we tested the macrophage response after co-culturing with heat-killed *mar1Δ* cells. Similar to live cells, we found that heat-killed *mar1Δ* cells also induce increased TNF-α production by BMMs, demonstrating that *mar1Δ* macrophage stimulation is not dependent on cell viability (Fig 8A).

Due to our observation that the *mar1Δ* cell wall changes are only induced when the cells are incubated in host-mimicking tissue culture conditions, we tested both YPD- and tissue culture media-incubated cells in our BMM assays. Consistent with the host-induced changes in the *mar1Δ* cell wall, only cells pre-incubated in TC medium prior to co-culture induced increased TNF-α production from BMMs (Fig 8C). This was the case for both live and heat-killed cells. We also examined whether the cell wall itself was sufficient to elicit a response from BMMs. Compared to WT and reconstituted strain controls, we observed that isolated cell walls from the *mar1Δ* strain pre-incubated in TC medium induced more TNF-α production by BMMs (Fig 8D). Together these data suggest that macrophage activation by this strain is dependent on specific cell wall changes that are induced by host-mimicking tissue culture conditions.

### Cell wall changes in *mar1Δ* increase recognition of the acapsular *cap59Δ* mutant

The polysaccharide capsule can serve to shield the immunogenic cell surface of *C*. *neoformans* from immune recognition. Additionally, capsule polysaccharide itself can be immunosuppressive [9]. We have observed that the *mar1Δ* mutant does not properly attach capsule to its cell surface, but it seems to secrete the polysaccharide similarly to WT (Fig 2A and 2B). Therefore, we wanted to determine if the capsule-deficient phenotype of *mar1Δ* is contributing to its ability to stimulate macrophages. To test this, we generated a *mar1Δ cap59Δ* double mutant; Cap59 is involved in capsule biosynthesis, and the *cap59Δ* mutant does not produce any detectable capsule polysaccharide [14,44]. We used the *cap59Δ* single mutant as our control strain, and tested macrophage activation of the *mar1Δ cap59Δ* double mutant compared to *cap59Δ* alone. We observed that the *mar1Δ cap59Δ* double mutant induced significantly more TNF-α than the single *cap59Δ* mutant (S5 Fig). This indicates that the cell surface of *mar1Δ* has a role in activating macrophages that is independent of this strain’s capsule defect and separate from any cell surface changes that the *cap59Δ* mutant exhibits as a result of its inability to produce capsular polysaccharide.

### Mar1 is required for full virulence *in vivo*

**Fig 9:**
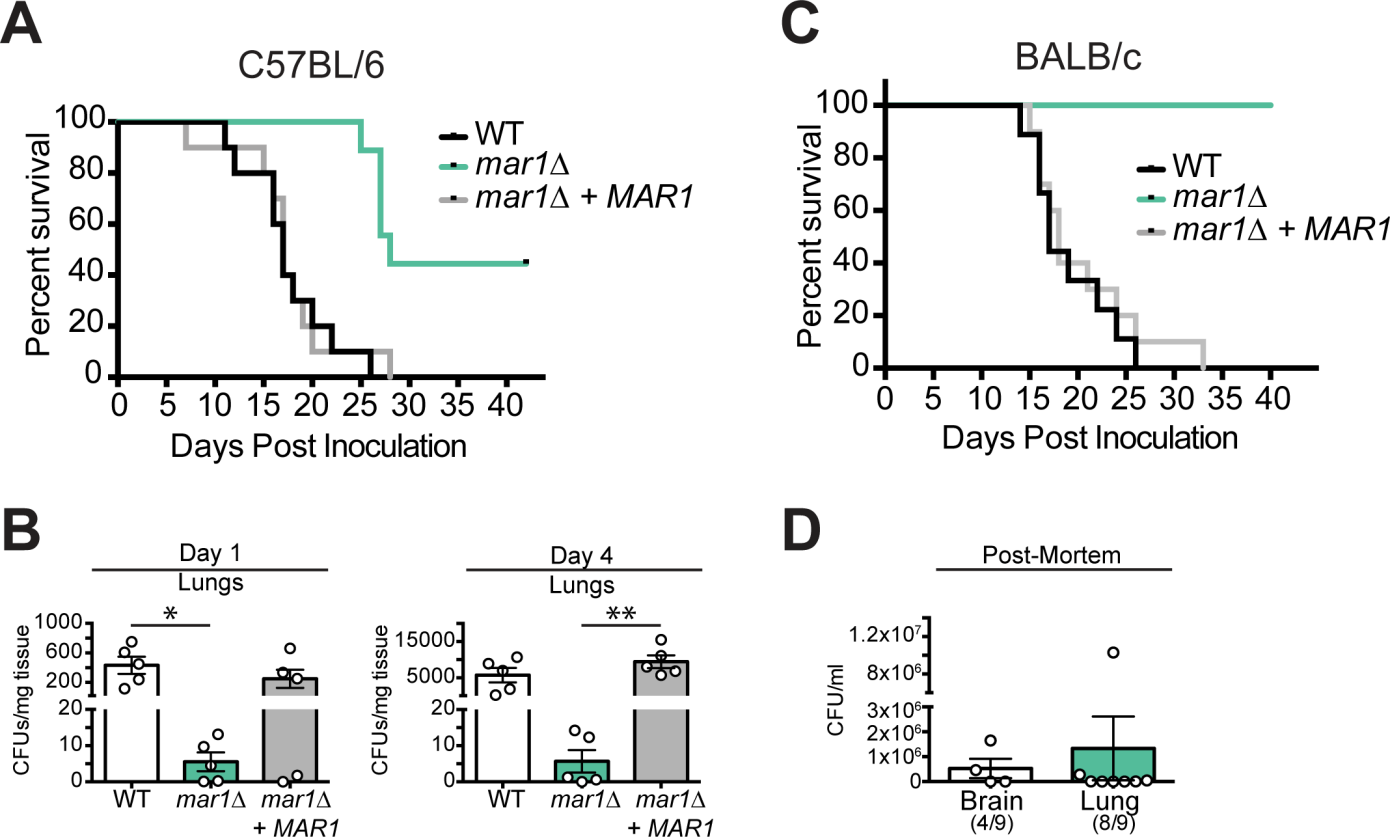
Mar1 is required for full virulence. (A) The *mar1Δ* strain is attenuated in the C57BL/6 mouse background. For each strain, 9–10 C57BL/6 mice were inoculated with 1 x 10^5^ cells, monitored daily for signs of infection, and sacrificed at predetermined clinical endpoints that predict mortality. Statistical significance was determined by log-rank test with Bonferroni correction: p < 0.0001, *mar1Δ* vs. WT; p < 0.001, *mar1Δ* vs. *mar1Δ + MAR1*; not significant. (B) There are minimal *mar1Δ* cells in the lungs of infected mice at early time points. Colony forming units (CFUs) were determined from lung homogenates from 5 mice per strain at days 1 and 4 post inoculation. *, p < 0.05 *mar1Δ* vs. WT; **, p < 0.01 *mar1Δ* vs. *mar1Δ* + *MAR1* as determined by one-way ANOVA with Tukey’s multiple comparisons test. (C) The *mar1Δ* strain is avirulent in the BALB/c mouse background. For each strain, 9–10 BALB/c mice were inoculated with 1 x 10^5^ cells, monitored daily, and sacrificed as described above. Statistical significance was determined by log-rank test with Bonferroni correction: p < 0.0001, *mar1Δ* vs. WT/*mar1Δ* + *MAR1*; not significant, WT vs. *mar1Δ* + *MAR1*. (D) The *mar1Δ* strain is not completely cleared from BALB/c mice despite mouse survival. CFUs were determined from lung and brain homogenates from *mar1Δ* infected mice post-mortem. Ratio indicates number of mice represented.

We used a murine inhalation model of cryptococcosis to assess the role of Mar1 in virulence [45]. We intranasally inoculated C57BL/6 mice with WT, *mar1Δ*, or *mar1Δ + MAR1* complemented cells and monitored mice over the course of 40 days for clinical endpoints predictive of mortality. Mice infected with the WT or *mar1Δ* + *MAR1* complemented strains exhibited a median survival time of 18 days (Fig 9A). In contrast, mice infected with the *mar1Δ* mutant had a median survival time of 28 days. We also measured fungal burden in the lungs at early time points after infection. As early as days 1 and 4 after infection, the number of *mar1Δ* mutant cells were significantly decreased in the lungs of infected mice compared to WT cells (Fig 9B). However, half of the *mar1Δ*-infected mice eventually succumbed to infection, suggesting there is not complete clearance of these cells.

It has been previously documented that mice from different genetic backgrounds display varying levels of sensitivity to cryptococcal infection [46,47]. C57BL/6 mice predominate towards protective Th1 type responses, while BALB/c mice have a non-protective Th2 type bias [48]. Therefore, we also tested the susceptibility of BALB/c mice to *mar1Δ* infection. We intranasally inoculated BALB/c mice as above and monitored mice over the course of a 40-day infection. In contrast to C57BL/6 mice, all of the *mar1Δ*-infected BALB/c mice survived the course of the experiment (Fig 9C). At the end of the experiment, we measured fungal burden in the lungs and brain post-mortem and observed that *mar1Δ* is not completely cleared from all mice, despite mouse survival (Fig 9D).

### *In vitro* response to *mar1Δ* requires members of the C-type lectin receptor and Toll-like receptor families

The initial innate interaction between fungi and the host begins with pathogen recognition by pattern recognition receptors (PRRs) on innate immune cells. Members of the C-type lectin receptor (CLR) and Toll-like receptor (TLR) families have been implicated in recognizing fungal pathogen-associated molecular patterns (PAMPs), including fungal cell wall components. Many of the CLRs signal through the adaptor protein, Card9, to downstream cellular pathways to activate pro-inflammatory cytokines. Similarly, several of the TLRs signal through the adaptor protein, MyD88, to activate downstream cellular responses.

**Fig 10:**
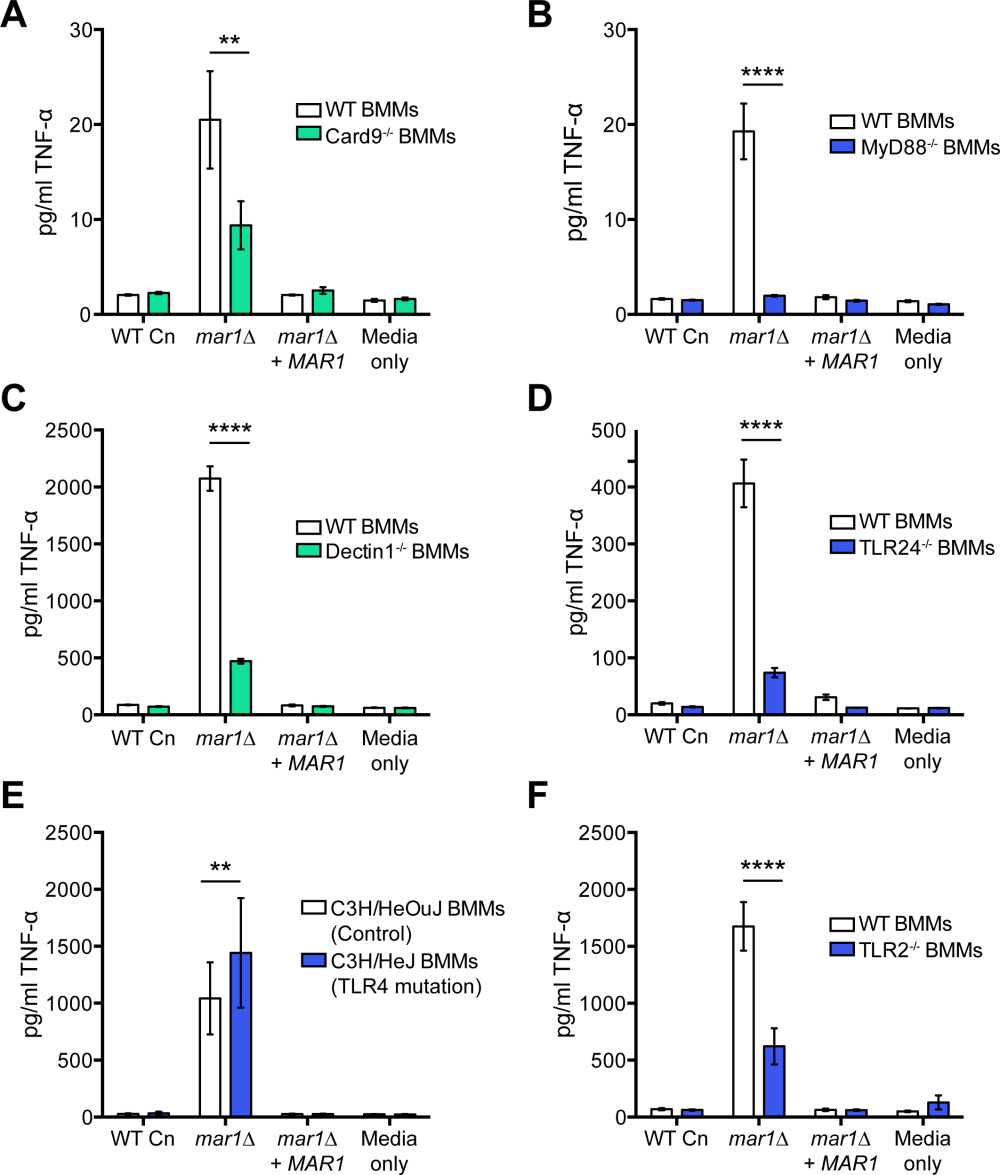
Macrophage activation in response to *mar1Δ* requires members of the CLR and TLR families. BMMs were harvested from the indicated mouse strains (C57BL/6 background, unless otherwise noted) and co-incubated with *C*. *neoformans* (Cn) cells at an MOI of 10:1, Cn:BMMs, followed by quantification of TNF-α (pg/ml) in the supernatant by ELISA. (A) Card9 is involved in macrophage activation by *mar1Δ* cells. Data represent means of 3 replicates from 3 independent experiments (n = 9). **, p < 0.01 WT vs. Card9^-/-^ BMMs as determined by two-way ANOVA with Sidak’s multiple comparisons test. (B) MyD88 is required for TNF-α production by macrophages in response to *mar1Δ*. Data represent means of 3 replicates from 3 independent experiments (n = 9). ****, p < 0.0001 WT vs. MyD88^-/-^ BMMs as determined by two-way ANOVA with Sidak’s multiple comparisons test. (C) Dectin-1 is involved in the response to *mar1Δ* cells. Data represent means of 3 replicates from 2 experiments (n = 6). ****, p < 0.0001 WT vs. Dectin-1^-/-^ BMMs as determined by two-way ANOVA with Sidak’s multiple comparisons test. (D) TLR2/4^-/-^ BMMs do not respond to *mar1Δ* cells. Data represent means of 3 replicates (n = 3). ****, p < 0.0001 WT vs. TLR2/4^-/-^ BMMs as determined by two-way ANOVA with Sidak’s multiple comparisons test. (E) TLR4 is not required for the production of TNF-α induced by *mar1Δ* cells. BMMs were isolated from C3H/HeJ mice with a null mutation in TLR4, and C3H/HeOuJ control mice. Data represent means of 3 replicates from 2 independent experiments (n = 6). **, p < 0.01 C3H/HeOuJ vs. C3H/HeJ BMMs as determined by two-way ANOVA with Sidak’s multiple comparisons test. (F) Macrophage activation by *mar1Δ* is partially dependent on TLR2. Data represent means of 3 replicates from 3 independent experiments (n = 9). ****, p < 0.0001 WT vs. TLR2^-/-^ BMMs as determined by two-way ANOVA with Sidak’s multiple comparisons test.

To determine if members of the CLR or TLR families are responsible for recognizing the *mar1Δ* cell surface, we examined the roles of the Card9 and MyD88 adaptor proteins in macrophage activation by these strains. We co-cultured our strains with BMMs isolated from Card9^-/-^ and MyD88^-/-^ mice and compared the production of TNF-α with BMMs isolated from WT mice. Consistent with our results above, the *mar1Δ* mutant induced significantly more TNF-α production from WT BMMs than isogenic WT *C*. *neoformans* strains. By contrast, the Card9^-/-^ BMMs had a significant, but incomplete, reduction in their response to the *mar1Δ* mutant, indicating a role for both Card9-dependent and–independent receptors (Fig 10A). Strikingly, the exaggerated TNF-α response to the *mar1Δ* strain was completely absent in MyD88^-/-^ BMMs, suggesting a significant role for TLRs in recognizing the *mar1Δ* cell surface (Fig 10B). Combined, these data demonstrate that both CLR and TLR family receptors are likely involved in sensing and responding to the *mar1Δ* strain.

### Recognition of the *mar1Δ* cell surface involves multiple PRRs important for chitin sensing

Based on the importance of both Card9 and MyD88 in sensing and responding to *mar1Δ* cells, we next focused on specific PRRs that have been previously implicated in fungal cell wall recognition, in particular chitin and chitooligomers. The C-type lectin receptor, Dectin-1 has been studied extensively for its role in recognizing the fungal cell surface, in particular β-glucan in the cell wall [49]. It has also been implicated in sensing fungal chitin and leading to a pro-inflammatory response, including the production of TNF-α, from innate immune cells [19,20]. To test the role of Dectin-1 in recognizing *mar1Δ* cells, we co-cultured our strains with Dectin-1^-/-^ BMMs and measured the production of TNF-α after 6 hours (Fig 10C). Compared to WT BMMs, the response to *mar1Δ* by Dectin-1^-/-^ BMMs was significantly decreased, indicating that this C-type lectin receptor is involved in sensing *mar1Δ*.

Due to the marked decrease in the *mar1Δ* response by MyD88^-/-^ BMMs, we used BMMs isolated from TLR2/4^-/-^ mice to focus on the extracellular TLRs that have been previously implicated in fungal PAMP recognition [50–52]. We co-cultured our strains with TLR2/4^-/-^ BMMs and measured the TNF-α response after 6 hours compared to WT BMMs. Similar to what we observed for MyD88^-/-^ BMMs, TLR2/4^-/-^ BMMs showed significantly reduced responses to *mar1Δ*, indicating a role for TLR2 and/or TLR4 in sensing this fungal cell surface (Fig 10D).

To test the individual role of TLR4, we utilized C3H/HeJ mice that have a null mutation in the TLR4 gene [53]. These mice have been used in several studies to assess the role of TLR4 in the response to a variety of microbial pathogens or pathogen products including bacteria (*Mycobacterium* [54], *Bordetella* [55], *Rickettsia* [56], *Neisseria* [57]) and fungi (*Coccidioides* [58], *Aspergillus* [59]). We isolated BMMs from C3H/HeJ mice and C3H/HeOuJ control mice and assessed their TNF-α responses to *mar1Δ*. We found a modest increase in the response to *mar1Δ* between C3H/HeJ TLR4 mutant BMMs and C3H/HeOuJ control mice (Fig 10E). In control experiments, both mutant and control BMMs responded similarly to a control ligand, zymosan; however, as expected, C3H/HeJ BMMs did not respond to the canonical TLR4 ligand, LPS, while C3H/HeOuJ BMMs responded normally (S6 Fig). These results suggest that the response to *mar1Δ* is TLR4-independent.

In previous work, TLR2 has been described to have a role in recognizing fungal chitin, leading to a pro-inflammatory response and the production of TNF-α [18–20]. To test if TLR2 is responsible for recognizing the *mar1Δ* surface, we carried out our co-culture assays using TLR2^-/-^ BMMs. The TNF-α response after 6 hours was significantly reduced in the TLR2^-/-^ BMMs compared to WT BMMs (Fig 10F). These data suggest that the decrease in response to *mar1Δ* in the TLR2/4^-/-^ BMMs was due to the defect in TLR2. Together these results support a model in which the TLR2 and Dectin-1 PRRs are both involved in sensing and responding to the *mar1Δ* cell surface.

## Discussion

The fungal cell wall is a dynamic structure that is constantly being remodeled. Fungal pathogens carefully regulate their cell wall in the context of the host in order to adapt to this environment, as well as to avoid detection by the immune system. Here we have identified a novel cell wall remodeling protein in *C*. *neoformans*, Mar1, and demonstrated that this protein has a role in intracellular trafficking that results in the proper localization of the cell wall β-(1,3)-glucan synthase, Fks1, as well as the continuous reorganization of the fungal cell surface in host-like conditions. The immunological consequence of this cell wall mis-regulation in the *mar1Δ* mutant is an increased activation of macrophages and dendritic cells as well as attenuated virulence *in vivo*.

**Fig 11:**
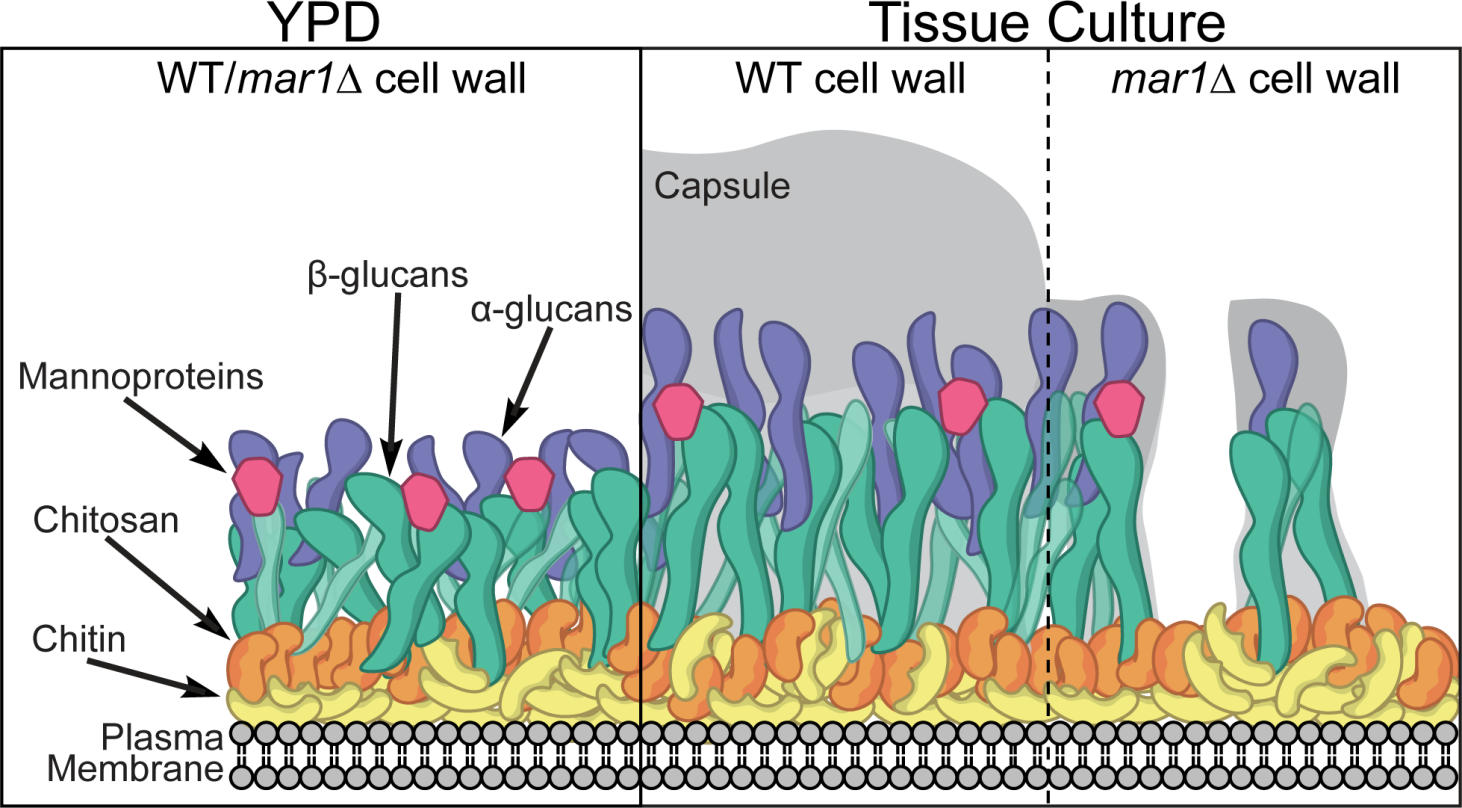
The cell surface of *C*. *neoformans* is remodeled in response to host-like conditions. When incubated in tissue culture medium, there is an increase in the expression of genes encoding the biosynthesis enzymes for outer cell wall components, resulting in a thicker cell wall, enhanced capsule attachment, and immune avoidance. The Mar1 protein is required for controlling aspects of these cell wall adaptations, including the proper induction and localization of glucan synthases. As a result, in the *mar1Δ* mutant the levels of these outer cell wall components are reduced, exposing the more immunogenic chitooligomers that are normally masked from immune recognition.

Our lab and others have demonstrated that the cell wall changes in size and in composition in response to the host environment [14–16,60]. Here, in WT cells, we have observed an increase in cell wall thickness in TC medium by TEM. This occurs in the setting of the well characterized formation and expansion of polysaccharide capsule. We have also measured increased expression of many of the known cell wall synthase genes in response to TC medium, and we have demonstrated that Fks1 is enriched at the plasma membrane in a specific population of WT cells after incubation in TC medium. By contrast, we observed a thickened, but less structurally sound cell wall in *mar1Δ* cells incubated in TC medium. Despite this observed increase in cell wall thickness, we showed that the *mar1Δ* strain has decreased total levels of glucans and mannans, decreased expression of glucan synthase genes, and decreased Fks1 protein at the plasma membrane in TC-incubated cells. We also demonstrate that *mar1Δ* cells do not properly attach capsule to their cell surface. Together these data suggest a model in which the *mar1Δ* strain has decreased total levels of outer cell wall components (glucans and mannans), leading to the observed capsule attachment defect and increased exposure of the inner cell wall components chitin and chitosan (Fig 11).

### Mar1 is impacting cell wall remodeling through regulation of the cell wall synthase response to host-like conditions

Our model demonstrates a prominent role for Mar1 in the cell wall response to TC medium, in particular through the regulation of cell wall synthase expression and localization. Previous studies in model fungi have shown differential localization of cell wall synthases in response to different conditions. The *S*. *cerevisiae* chitin synthase, Chs3, is maintained under normal conditions in internal compartments called “chitosomes”, where it is cycled between early endosomes and the trans-Golgi network in a clathrin adaptor protein (AP) complex dependent manner [61]. However, under stress conditions, Chs3 is trafficked to the plasma membrane in order to synthesize increased chitin [62]. Similar clathrin AP complex-dependent trafficking has been described for the *Schizosaccharomyces pombe* β-(1,3)-glucan synthase, Bgs1. Mutations in the conserved AP-1 adaptor complex protein, Sip1, and the clathrin light chain protein, Clc1, lead to defects in Bgs1 localization/delivery to the plasma membrane [63,64].

Here we have observed that Fks1 plasma membrane localization is enriched in specific WT cell populations upon shifting cells to tissue culture medium, suggesting a similar stress-induced cycling of this protein to its functional location at the cell surface. Mar1 could be impacting the cycling of Fks1 between internal compartments and the plasma membrane in multiple ways. For example, Mar1 may be required for exocytic movement towards the plasma membrane, and therefore in the absence of this protein, exocytosis and/or secretion of Fks1 is defective. However, the fact that capsule, melanin, and acid phosphatase secretion remain intact in this strain would suggest that Mar1 is not impacting generalized exocytosis or secretion. Another way in which Mar1 could be impacting Fks1 trafficking is if it were required for protein maintenance on the PM, for example, by preventing excessive endocytosis or recycling. Our data showing altered endocytosis of FM4-64 in the *mar1Δ* strain would support this model.

Another alternative mechanism by which Mar1 might impact Fks1 localization and ultimately cell wall remodeling could be through a role in cell surface signaling. The Mar1 protein has two transmembrane domains and our microscopy data indicates that it localizes to cellular membranes at the cell surface. Therefore, it is feasible to hypothesize that Mar1 is serving a signaling role, perhaps as a cell surface receptor, propagating the cue to traffic cell wall synthase proteins from internal stores to the PM under stress conditions. This model for Mar1 function, unlike the previous, would also explain the lack of transcriptional response of the *FKS1* and *AGS1* genes in the *mar1Δ* background. If the mutant cells are unable to sense specific stress conditions, it is plausible that they would not induce transcription of the enzymes required to respond to that stress.

The differential localization of Mar1 in YPD and TC media is also consistent with Mar1 serving a sensor/receptor role. We observed many Mar1 puncta, particularly on the cell surface, but also on intracellular membranes resembling the endoplasmic reticulum when the cells were incubated in YPD medium. By contrast, when shifted to TC medium, we observed a decrease in the number of puncta, as well as changes in their size/shape. It is possible that this decrease in puncta represents the endocytosis of Mar1 from the membrane to transmit a signal downstream, or perhaps internalization for degradation. Interestingly, we observed these changes in Mar1-localization at similar time points as increases in cell wall chitooligomer staining in the *mar1Δ* mutant; these observations suggest a correlation between Mar1 localization and the resulting cell wall response.

A role for Mar1 as a sensor implies a particularly intriguing model, as few cell surface stress sensors have been identified by sequence homology in *C*. *neoformans*. Interestingly, the putative Rra1 pH sensor was recently identified in *C*. *neoformans*; while appearing structurally and functionally similar to that of the pH sensors in ascomycete fungi, it was found to share no significant sequence similarity [35]. While Mar1 does not share sequence similarity with any known sensor proteins, its structural features and pattern of dynamic localization suggest that it could be serving as a functional homologue of a cell stress sensor.

### Proper cell wall regulation is required for effective immune avoidance

The importance of cell wall remodeling, in particular as it relates to cell wall masking, has been well-described in other fungi. The α-glucan layers of *H*. *capsulatum* and *A*. *fumigatus* have both been implicated in shielding β-glucan in these fungi. β-glucanases that degrade exposed β-glucan have also been elucidated in *H*. *capsulatum* [65]. In *C*. *albicans*, several factors have been implicated in β-glucan exposure including morphotype switching, antifungal exposure, phospholipid production, and carbon source [5,6,66]. For example, Ballou, *et al*. recently demonstrated that lactate exposure elicits increased β-glucan masking in *C*. *albicans* and other pathogenic *Candida* species, leading to reduced recognition by immune cells [6]. Furthermore, cell wall remodeling has been shown to drive immune function even after ingestion by host phagocytes. O’Meara, *et al*. showed that *C*. *albicans* actively remodels its cell surface upon phagocytosis in order to induce inflammatory cell death pathways in macrophages to aide in fungal cell escape [15].

*C*. *neoformans* is particularly adept at hiding from the immune system. Importantly, the polysaccharide capsule serves to effectively shield potentially immunostimulatory molecules from host detection [29,67]. However, there are several examples of the immune consequences of improper cell wall organization in *C*. *neoformans*. The loss of α-(1,3)-glucan results in a highly disorganized cell wall, with increased chitin and chitosan content and redistributed β-glucan, rendering cells avirulent in mouse models of infection [68]. Chitosan-deficient strains of *C*. *neoformans* are also unable to cause disease in mice, inducing a protective proinflammatory host immune response that leads to their rapid clearance [69–71]. Imprecise regulation of the cell wall has also been implicated in excessive immune responses related to improper *C*. *neoformans* cell wall exposure, in particular chitooligomers (chitin and/or chitosan). Our laboratory has previously studied the importance of the Rim101 alkaline pH transcription factor in regulating cell wall remodeling [14–16]. The *rim101Δ* mutant exposes increased cell wall chitooligomers, which results in an excessive and nonprotective immune response *in vivo* [16]. Likewise, Wiesner, *et al*. demonstrated that strains with increased chitin abundance induced unfavorable immune outcomes and exacerbated disease [12].

Here, we propose that both the exposure and total levels of cell wall components are important determinants of immune recognition of fungi. Based on our HPLC data, our model suggests that *mar1Δ* has less β-glucan in its cell wall (Fig 11). Therefore, one might predict that immune signaling would be less active with reduced immunostimulatory β-(1,3)-glucan to sense. However, the *mar1Δ* cell wall has more exposed chitooligomers than WT. As noted above, several studies have highlighted the importance of chitin and chitin-derived structures in *C*. *neoformans* immune recognition [12,16]. Additionally, cells in which chitooligomer exposure was blocked by WGA exhibited reduced association with murine macrophages [72]. While a single chitin receptor has not been identified to date, several PRRs have been implicated in different aspects of chitin recognition. Here we have demonstrated that multiple PRRs are also involved in the recognition of the *mar1Δ* cell wall. Macrophage activation by *mar1Δ* was partially dependent upon Card9, the adaptor protein required for most C-type lectin receptor signaling, and entirely dependent on MyD88, the adaptor protein required for signaling through many Toll-like receptors (TLRs). Accordingly, we demonstrated a requirement for Dectin-1 and TLR2 in the activation of macrophages in response to the *mar1Δ* mutant strain. There are several possibilities as to why more than one PRR might be involved in this process. In addition to their roles individually, PRRs can function together to induce downstream immune signaling pathways. In particular, several groups have demonstrated a collaborative role between Dectin-1 and TLR2 in detecting fungal epitopes [73–75].

Wagener and colleagues proposed a model in which chitin recognition occurs in different stages during *C*. *albicans* interaction with immune cells [20]. During early interaction, chitin on the surface of this fungus is recognized by Dectin-1 and TLR2, resulting in the secretion of proinflammatory TNF-α. Over time this leads to the secretion of chitinases and a decrease in pathogen load. Later in this interaction, digested fragments of chitin are phagocytosed by immune cells and recognized by intracellular TLR9 and NOD2 in a mannose receptor (MR)-dependent manner, resulting in the secretion of IL-10. This latter state occurs during the resolution of infection. In agreement with this model, other groups have demonstrated that intermediate-sized chitin stimulated macrophage production of TNF-α that is dependent on TLR2, Dectin-1, and MR. In contrast, small chitin fragments induced IL-10 production [19]. Given the importance of chitooligomer size and structure in the immunostimulatory process, future work on the mechanism of chitin and chitosan synthesis and degradation will elucidate its role in fungal immune recognition and evasion. Our data demonstrating that intact *mar1Δ* cells induce increased TNF-α from macrophages in a TLR-2 and Dectin-1 dependent manner support these emerging models of the centrality of chitooligomer exposure in fungal stimulation of the host immune system.

It is still possible, that other factors are contributing to the *mar1Δ* macrophage activation phenotype. While we demonstrated decreased overall levels of glucans and mannans in *mar1Δ* cell wall extracts, this does not necessarily exclude these components from playing a role. We did not observe a significant signal from Fc-Dectin-1 labeled cells above baseline, however we did see a very modest increase in *mar1Δ* cells stained with the α-glucan antibody, MOPC-104E, and the mannoprotein-binding lectin, Concanavalin A (Fig 4A). While α-glucans generally serve to mask other cell wall components, *Cryptococcal* mannoproteins are well-known to illicit immune responses [76]. We have also shown that *mar1Δ* cells lack a polysaccharide capsule, and further demonstrated that this is due to a capsule attachment defect, rather than a biosynthesis or secretion defect. Polysaccharide capsule serves to shield the cell surface from immune detection, and free capsule has been shown to have immunomodulatory effects [9,77]. Using a *mar1Δ cap59Δ* double mutant, in which capsule biosynthesis is inhibited, we showed that the *mar1Δ* cell wall has an impact on macrophage activation that is independent of the *cap59Δ* single mutant strain, suggesting this response is not due simply to the lack of capsule in the *mar1Δ* background. We also determined that heat killed *mar1Δ* cells, in which capsule polysaccharide is not being actively shed, are still immunostimulatory.

Based on our data, we suggest multiple ways in which this capsule defect may arise. First, it has been previously shown that capsule attachment to the cell wall requires Ags1 expression and α-glucan [13]. We demonstrated that the *mar1Δ* mutant has decreased glucans in its cell wall and severely decreased Ags1 expression. We also observed that while *mar1Δ* sheds a similar amount of capsule polysaccharide into the media as WT cells, it migrated at a different rate. This could suggest that the major capsule polysaccharide component, GXM, is modified in some way as to inhibit its ability to attach to the cell wall.

Additional yet unidentified PRRs may also be involved in this interaction. In addition to further interrogating the role of mannoproteins in the *mar1Δ* response, future work will examine the importance of the mannose receptor in this interaction. The role of TLR9 and NOD2 in this interaction is also intriguing. We did not explore these intracellular receptors in this study, however TLR9 and NOD2 have been previously implicated in recognizing fungal chitin that has been digested and phagocytosed by mammalian macrophages [20]. This recognition leads to the production of the anti-inflammatory cytokine, IL-10, and is thought to lead to the resolution of the pro-inflammatory immune response to intact fungal chitin [20]. In earlier studies, it was observed that, in fact, TLR9^-/-^ macrophages co-incubated with *C*. *albicans* and *S*. *cerevisiae* had increased TNF-α production, suggesting a role for TLR9 in modulating the pro-inflammatory response to these fungi [78]. TLR9 deficiency has also been associated with worsened immune outcomes in *C*. *neoformans* [79,80].

### Conclusions

In summary, we have identified a novel cell wall regulatory protein, Mar1. This protein, while not apparent in any canonical cell wall regulatory pathway, is required for proper cell wall organization in host-like conditions. The Mar1 protein localizes to discrete puncta on cellular membranes, with an apparent reduction in puncta upon shifting cells to host-like conditions. In the absence of Mar1, transcription of the α- and β-glucan synthases is not induced, leading to a decrease in these cell wall components and an increase in the exposure of the chitooligomers, chitin and chitosan. We propose that the role of Mar1 in cell wall integrity is in orchestrating the proper response to host-like conditions, occurring in part at the level of intracellular trafficking and results in the mis-localization of the β-(1,3)-glucan synthase, Fks1. Here we have also shown that this dysregulated cell wall manipulates the host-pathogen interaction, leading to increased macrophage activation that is dependent on multiple pattern recognition receptors. Finally, Mar1 is required for full virulence in two mouse models of systemic cryptococcosis.

## Materials and methods

### Strains, media, and growth conditions

*Cryptococcus neoformans* strains used in this study are listed in Table 1. Unless otherwise noted, all strains were generated in the *C*. *neoformans* var. *grubii* H99 background and maintained on YPD agar plates (2% yeast extract, 1% peptone, 2% dextrose). Strains created by crosses were co-incubated on MS mating media [81], followed by spore isolation by microdissection. Recombinant spores were identified by PCR and selectable marker resistance. To assess cell wall associated phenotypes, NaCl (1.5 M) and Congo red (0.5%) were added to YPD medium prior to autoclaving; caffeine (1 mg/ml) and calcofluor white (1 mg/ml) were filter sterilized and added to YPD after autoclaving. Alkaline pH plates were made by adding 150 mM HEPES buffer to YPD and adjusting the pH (pH 8.15) with NaOH prior to autoclaving. To induce and visualize capsule, strains were incubated in CO_2_-independent tissue culture medium (TC, Gibco) for 72 hours with shaking at 37°C, followed by staining with India Ink.

**Table 1:** *C*. *neoformans* strains used in this study.

Strain	Genotype	Source
H99	*MAT*α	[82]
KN99a	*MAT***a**	[83]
SJB12	*MAT*α *mar1*^*ΔT-DNA*^	This study
MAK1	*MAT*α *mar1Δ*::*NAT*	This study
MAK11	*MAT*α *mar1Δ*::*NAT + MAR1-NEO*	This study
TOC35	*MAT*α *rim101Δ*::*NAT*	[16]
TOC97	*MAT*α *pka1Δ*::*NAT*	[15]
KK3	*MAT***a** *mpk1Δ*::*NAT-STM#150*	[84]
KK6	*MAT*α *cna1Δ*::*NAT-STM#177*	[84]
YSB64	*MAT*α *hog1Δ*::*NAT-STM#177*	[85]
SKE106	*MAT*α *mar1Δ*::*NAT + HGFP-MAR1-NEO*	This study
KS208	*MAT***a** *rim101Δ*::*NAT + eGFP-RIM101*	[35]
MAK8	*MAT*α *mar1Δ*::*NAT* + e*GFP-RIM101*	This study
SKE94	*MAT*α *MPK1-4FLAG-NEO*	This study
SKE96	*MAT*α *mar1Δ*::*NAT + MPK1-4FLAG-NEO*	This study
SKE87	*MAT*α *mar1Δ*::*NEO mpk1Δ*::*NAT*	This study
KMP13	*MAT*α *FKS1-GFP-NEO*	This study
CLT1	*MAT*α *FKS1-GFP-NEO mar1Δ*::*NAT*	This study
CLT2	*MAT*α *FKS1-GFP-NEO mar1Δ*::*NAT*	This study
*cap59Δ*	*MAT*α *cap59Δ*::*NEO*	[14]
CBN377	*MAT***a** *cap59Δ*::*NEO*	[14]
SKE60	*MAT*α *mar1Δ*::*NAT cap59Δ*:*NEO*	This study

For cell wall staining, cell wall isolation, protein localization microscopy, FM4-64 staining, and *in vitro* co-culture experiments, overnight YPD cultures were diluted 1:10 in YPD liquid medium (at 30°C) or in TC medium (at 37°C) for 16–18 hours with shaking (150 rpm), unless otherwise noted. These methods were described previously [16].

### Molecular biology

All primers used in this study are listed in Table 2. All plasmids used in this study are listed in Table 3. The *mar1*^*ΔT-DNA*^ strain was generated by *Agrobacterium* tumefaciens-mediated transformation (AMT) as described previously [22]. *C*. *neoformans* targeted gene deletion strains were generated by homologous recombination to replace the entire open reading frame (ORF) with a dominant selectable marker. The deletion cassettes were created using overlap PCR as described previously [86,87] using the primers indicated in Table 2. Deletion cassettes were introduced into the indicated background strain by biolistic transformation [88]. All deletion strains were confirmed by PCR and Southern blot; primers used to design probes for Southern blot can be found in Table 2.

**Table 2:** Primers used in this study.

Primer name	Primer sequence (5’-3’)	Purpose
*Deletion Cassettes*		
AA3640	GAAGAGGGCAATAAAGGAGA	*mar1Δ* primer 1
AA3641	GTCATAGCTGTTTCCTGTTGAGGACAGTGACGGTTGGACA	*mar1Δ* primer 2
AA3642	TGTCCAACCGTCACTGTCCTCAACAGGAAACAGCTATGAC	*mar1Δ* primer 3
AA3643	AACTATTGACCTCTTCTTAGTTTTCCCAGTCACGACG	*mar1Δ* primer 4
AA3644	CGTCGTGACTGGGAAAACTAAGAAGAGGTCAATAGTT	*mar1Δ* primer 5
AA3645	TATAACGAAGGGGCATGATA	*mar1Δ* primer 6
AA4096	AAGGTGTTCCCCGACGACGAATCG	NAT split marker
AA4097	AACTCCGTCGCGAGCCCCATCAAC	NAT split marker
AA3934	TCGATGCGATGTTTCGCT	*NEO* split marker
AA3935	CCTGAATGAACTGCAGGA	*NEO* split marker
*Southern probes*		
AA3670	TTGCGGGAAGACCTTCACTA	*MAR1* Southern probe F
AA3671	GCTGCGTTCGCACTGTACTA	*MAR1* Southern probe R
AA4903	TCCCCTTCGACTTTTCCTTT	*MPK1-4FLAG* Southern probe F
AA4904	ATGTTGAGGTGCAGGAGGAG	*MPK1-4FLAG* Southern probe R
*Cloning*		
AA4894	GTACGAGCTCGGATCCATGGCCGCTTTCGACCTA	*MAR1-GFP-NEO* Frag F
AA4895	CGTTACTAGTGGATCCCTTTCAGATTACCTTCAACTA	*MAR1-GFP-NEO* Frag R
AA4829	CGGTACCCGGGGATCCCCGTTGTATCCTAACGC	*MPK1-4FLAG* Frag 1 F
AA4830	ATCTGGCGCGCCAGGTGATAATTTCTGCCTCTCCA	*MPK1-4FLAG* Frag 1 R
AA4831	CCTGGCGCGCCAGATTAC	*MPK1-4FLAG* Frag 2 F
AA4832	TAATACAGATAAACCCCTCAATCTATCCCTCTCT	*MPK1-4FLAG* Frag 2 R
AA4264	GGTTTATCTGTATTAACACG	*MPK1-4FLAG* Frag 3 F
AA4598	TCGACAAATATGATTGCTGCGAGGATGTGAGCT	*MPK1-4FLAG* Frag 3 R
AA4599	AATCATATTTGTCGAGTCTGT	*MPK1-4FLAG* Frag 4 F
AA4833	TGATTACGCCAAGCTGAAGGAATTATGCTGTGGTC	*MPK1-4FLAG* Frag 4 R
AA4545	TCGGTACCCGGGGATCCCTCACAGCTGAACTC	*FKS1-GFP-NEO* Frag 1 F
AA4362	CGCCCTTGCTCACCATGATGATACCGTTGAAAGGC	*FKS1-GFP-NEO* Frag 1 R
AA4364	ATGGTGAGCAAGGGCG	*FKS1-GFP-NEO* Frag 2 F
AA4400	GATAACGCTCGGTACCTAGTACAGCTCGTCCATG	*FKS1-GFP-NEO* Frag 2 R
AA4553	GTACCGAGCGTTATC	*FKS1-GFP-NEO* Frag 3 F
AA4426	GTTAATACAGATAAACCGTGACATGTAATTCGACG	*FKS1-GFP-NEO* Frag 3 R
AA4264	GGTTTATCTGTATTAACACG	*FKS1-GFP-NEO* Frag 4 F
AA1668	GCTGCGAGGATGTGAGCTG	*FKS1-GFP-NEO* Frag 4 R
AA4433	CTCACATCCTCGCAGCCCTCATAAGCCTCGTGGTAG	*FKS1-GFP-NEO* Frag 5 F
AA4546	GGTCGACTCTAGAGGATCCGATCACCTCCAACG	*FKS1-GFP-NEO* Frag 5 R
*Real-time PCR*		
AA301	AGTATGACTCCACACATGGTCG	*GPD1 F*
AA302	AGACAAACATCGGAGCATCAGC	*GPD1 R*
AA4298	ACCCAGGTCTGGCATTCC	*CHS3 F*
AA4299	AGGATCAACATTGGAAGC	*CHS3 R*
AA3628	CGGTCTTCAGGCATTGATTT	*CHS4 F*
AA3629	TTCGGAGTGAAGTGATGCTG	*CHS4 R*
AA4905	TTGACCCTTGGCACATCT	*CHS6 F*
AA4906	GTTGGCATAAGTATCCTT	*CHS6 R*
AA3632	TCGAGCTATTGCTGCTCAGA	*CDA1 F*
AA3633	GCTGGTAGATGTCGTGCTCA	*CDA1 R*
AA4304	GTAACGAGGTCGTCTTTG	*CDA2 F*
AA4305	TGTAGTTGGTGAGCTCGT	*CDA2 R*
AA3652	ATGTGGCCGATGCTTTTAAC	*CDA3 F*
AA3653	GAAGTGAGAAGGCCTGTTGG	*CDA3 R*
AA3828	ATCCTTATCCGTTATTCC	*AGS1 F*
AA3829	AGCTGTTCCTCTAGCGAGC	*AGS1 R*
AA3626	TGGACTGGTGTTTGGTTCAA	*FKS1 F*
AA3830	GTACAAAAGACCGTACTTG	*FKS1 R*
AA3654	GTCTCGGAAGGCGACTCAT	*KRE6 F*
AA3655	TCAACTCATTCTTTGGGAAGG	*KRE6 R*
AA3634	CTGGACAATGTATGCGGATG	*SKN 1 F*
AA3635	TCCGCAGTGGGATAATCTTC	*SKN 1 R*

**Table 3:** Plasmids used in this study.

Plasmid	ORF	Backbone	Source
pJAF	Neomycin resistance cassette (*NEO*)		[89]
pCH233	Nourseothricin resistance cassette (*NAT*)		[90]
pCN50	Histone H3 promoter; GFP	pJAF	[21]
pSKE26	Histone H3 promoter; GFP; *MAR1*, including terminator	pCN50	This study
pSKE19	3’ *MPK1*; C-terminal 4xFLAG; *HOG1* terminator; *NEO*; 3’ *MPK1* flaking region	pUC19	This study
pKP6	3’ *FKS1*; C-terminal GFP; *FKS1* terminator; *NEO*; 3’ *FKS1* flanking region	pUC19	This study

The *mar1Δ* deletion strain (MAK1) was constructed by replacing the *MAR1* ORF with the nourseothricin *(NAT*) cassette [90]. The *mar1Δ + MAR1* complemented strain (MAK11) was constructed by co-transformation of the WT *MAR1* allele with the pJAF neomycin (*NEO*) resistance vector into the MAK1 background.

The GFP-Mar1 strain (SKE106) was constructed by transforming pSKE26 into the *mar1Δ* (MAK1) background. The pSKE26 plasmid contains an N-terminally GFP-tagged Mar1 protein under the control of the histone H3 promoter. A fragment consisting of the *MAR1* open reading frame and ~ 500 bp of the 3’ UTR/terminator sequence was amplified from H99 genomic DNA using primer pair AA4894/AA4895. Using InFusion cloning (Clontech), this fragment was cloned in frame into the pCN50 backbone at a BamHI site at the end of GFP. Transformants were screened by wet colony morphology on pH 8 and GFP-Mar1 fusion was confirmed by PCR and western blot.

The *mar1Δ* + *eGFP-RIM101* (MAK8) strain was constructed by crossing MAK1 with KS208. Recombinant spores were screened by epifluorescent microscopy and confirmed by PCR.

The *MPK1-4FLAG-NEO* strains (SKE94 and SKE96) were generated by transforming pSKE19 into the WT or *mar1Δ* (MAK1) background. The *MPK1-4FLAG-NEO* tagging construct was designed such that a C-terminal 4xFLAG epitope tag would homologously recombine into the 3’ end of the *MPK1* locus. The pSKE19 plasmid was generated by In-Fusion cloning (Clontech) the following fragments into the pUC19 backbone: (1) ~500 bp of the 3’ end of *MPK1* ORF, amplified from H99 genomic DNA using primer pair AA4829/AA4830; (2) 4xFLAG linked to the *HOG1* terminator, amplified from pSG27 [91] using primer pair AA4831/AA4832; (3) *NEO* resistance cassette amplified from pJAF using primer pair AA4264/AA4598; (4) ~ 1 kb 3’ *MPK1* flank amplified from H99 genomic DNA using primer pair AA4599/AA4833. Transformants were screened by PCR and confirmed by Southern blot.

The *mar1Δ mpk1Δ* (SKE87) strain was constructed by replacing the *MAR1* ORF with the *NEO* cassette in the *mpk1Δ* deletion background (KK3). Transformants were screened by dry colony morphology on pH 8 and PCR.

The *FKS1-GFP-NEO* strain (KMP13) was constructed by transforming pKP6 into the WT background. The *FKS1-GFP-NEO* tagging construct was designed to facilitate homologous recombination at the *FKS1* locus. The pKP6 plasmid was created by In-Fusion cloning the following fragments into the pUC19 backbone: (1) ~ 1 kb of the 3’ end of the *FKS1* ORF, amplified from H99 genomic DNA using primer pair AA4545/AA4362; (2) *GFP* amplified from pCN19 (Price 2008) using primer pair AA4364/AA4400; (3) *FKS1* terminator (464 bp) amplified from H99 genomic DNA using primer pair AA4553/AA4426; (4) *NEO* resistance cassette amplified from pJAF using primer pair AA4264/AA1668; (5) 1 kb 3’ *FKS1* flank amplified from H99 genomic DNA using primer pair AA4433/AA4546. Transformants were screened by PCR and epifluorescent microscopy and integration into the locus was confirmed by PCR. The *mar1Δ FKS1-GFP-NEO* strains (CLT1 and CLT2) were generated by replacing the *MAR1* ORF with the *NAT* cassette in the KMP13 background. Transformants were screened by dry colony morphology on pH 8 and confirmed by PCR and Southern blot.

The *mar1Δ cap59Δ* (SKE60) strain was created by crossing MAK1 with CBN377. Recombinant spores were screened and confirmed by PCR.

### Capsule blot

The relative amount of capsule shedding in the cell supernatant was assayed as previously described [21,30]. Briefly, capsule induced cultures (incubated as described above) were incubated at 70°C for 15 minutes to denature enzymes, after which the cells were pelleted and the supernatant was sterile filtered. This conditioned medium was then run on a 0.6% agarose gel for 15 hours at 25 V, followed by transfer to a positively charged nylon membrane using Southern blotting methods. The membrane was air dried overnight, followed by blocking with 5% skim milk in Tris-Buffered Saline-Tween-20 (TBST). To detect capsule polysaccharide, blots were incubated with a mouse monoclonal anti-GXM antibody, MAb18B7 (1 μg/ml) [92,93] for 1 hour, washed 3x with TBST, and incubated with an anti-mouse horseradish peroxidase-conjugated secondary antibody (Jackson ImmunoResearch) for 1 hour. Blots were washed 3x with TBST and capsule polysaccharide was detected by enhanced chemiluminescence (ECL Prime Western blotting detection reagent; GE Healthcare).

### Cell wall staining and flow cytometry

Prior to all cell wall staining, cells were pelleted and washed 1-2x with phosphate buffered saline (PBS). For quantification by microscopy, stained cells were imaged on a Zeiss Axio Imager.A1 fluorescence microscope equipped with an AxioCam MRm digital camera (60X objective). The same exposure time was used to image all strains with the same stains. The mean gray value (sum of gray values for all the pixels in a cell divided by the number of pixels that make up the cell) of at least 100 cells was calculated using ImageJ/Fiji [94,95]. Results are reported as mean fluorescence values +/- standard error of the means.

For flow cytometry, cells were fixed with 3.7% formaldehyde for 5 minutes at room temperature, followed by washing 2x with PBS. Eosin Y stained samples were fixed with 10 mM sodium azide for 10 minutes at room temperature, followed by washing 2x with PBS. A total of 10^7^ cells/ml were stained and 10^6^ cells/ml were submitted to the Duke Cancer Institute Flow Cytometry Shared Resource for analysis using a BD FACSCanto II flow cytometer. Data was analyzed using FlowJo v10.1 software (FlowJo, LLC). Relevant events were gated in the forward scatter/side scatter (FSC/SSC) plots and then represented as histograms with mean fluorescence intensity (MFI) on the x-axis and cell counts on the y-axis. Unstained cells and cells incubated with secondary antibodies alone were used as negative controls.

To visualize chitin, cells were stained with 100 μg/ml FITC-conjugated wheat germ agglutinin (WGA; Molecular Probes) for 35 minutes in the dark, followed by 25 μg/ml calcofluor white (CFW) for 10 minutes. Prior to analysis, cells were washed 2x and resuspended in PBS. For microscopy, WGA was imaged using a GFP filter and CFW was imaged using a DAPI filter. For flow cytometry, WGA cells were analyzed using a 488 nm laser and CFW cells were analyzed using a 405 nm laser.

To visualize chitosan, sodium azide fixed cells were washed 2x with McIlvaine’s buffer (0.2 M Na_2_HPO_4_, 0.1 M citric acid, pH 6.0), followed by staining with 300 μg/ml Eosin Y (EY) in McIlvaine’s buffer for 5 minutes at room temperature. Prior to analysis, cells were then washed 2x and resuspended in McIlvaine’s buffer. For microscopy cells were visualized using a GFP filter. For flow cytometry cells were analyzed using a 488 nm laser.

The MOPC-104E antibody (Sigma) was used to visualize α-glucan, as described previously [3,14]. Briefly cells were incubated with 400 ng/ml MOPC-104E primary antibody for 1 hour, washed 2x with PBS, and incubated with 4 μg/ml anti-mouse AlexaFluor 488 secondary antibody (Jackson ImmunoResearch) for 30 minutes in the dark. Cells were washed 2x and resuspended in PBS prior to analysis. For flow cytometry cells were analyzed using a 488 nm laser.

An Fc-Dectin-1 fusion protein was used to visualize β-glucan (a gift from Gordon Brown, University of Aberdeen) [96–99]. Cells were resuspended in FACS block (0.5% BSA, 5% HI-rabbit serum, 5 mM EDTA, 2 mM NaAzide in PBS) for 10 minutes, followed by incubation with 5 μg/ml Fc-Dectin-1 protein for 40 minutes on ice. Cells were washed 3x with PBS and resuspended in 3.75 μg/ml anti-human AlexaFluor 488 secondary antibody (Jackson ImmunoResearch) in FACS wash (0.5% BSA, 5 mM EDTA, 2 mM NaAzide in PBS) for 30 minutes on ice. Cells were washed 2x and resuspended in PBS prior to analysis. For flow cytometry cells were analyzed using a 488 nm laser.

Concanavalin A conjugated to AlexFluor 488 (ConA; Molecular Probes) was used to visualize mannoproteins. Cells were resuspended in 50 μg/ml ConA for 1 hour, then washed 2x and resuspended in PBS prior to analysis. For flow cytometry cells were analyzed using a 488 nm laser.

### Microscopy

Differential interference microscopy (DIC) and fluorescent images were visualized with a Zeiss Axio Imager.A1 fluorescence microscope (60X or 100X objectives). Images were taken with an AxioCam MRm digital camera with ZEN Pro software (Zeiss). High-resolution fluorescent images were taken using a DeltaVision Elite deconvolution microscope equipped with a CoolSnap HQ2 high-resolution charge-coupled-device (CCD) camera. Images were processed using softWoRx software (GE). Images taken on both microscopes were additionally analyzed using ImageJ/Fiji software [94,95].

### Cell wall isolation and analysis

Chitin and chitosan levels were quantified using a modified MBTH (3-methyl-benzothiazolinone hydrazine hydrochloride) method as previously described [16]. Cell wall isolation and high performance anion-exchange chromatography with pulsed amperometric detection (HPAEC-PAD) were performed as previously described [16,33].

### RNA extraction and real time PCR analysis

Cells from an overnight YPD culture were washed 1x with water, diluted to 10^7^ cells/ml in YPD or TC medium in duplicate and incubated for 1.5 hours at 30°C (YPD) or 37°C (TC). Cultures were spun down and flash frozen on dry ice, followed by lyophilization. RNA was extracted using the RNeasy Plant Mini Kit (Qiagen) with optional on-column DNAse digestion. cDNA for real time-PCR (RT-PCR) was prepared using the AffinityScript cDNA synthesis kit (Agilent) with oligo(dT) primers. For RT-PCR, cDNA was diluted 1:3 in RNase-free water, added to IQ SYBR Green Supermix (Bio-Rad) per protocol instructions, and analyzed on an iCycler iQ Real-Time PCR Detection System (Bio-Rad). *GPD1* was used as an internal control, and negative control samples without reverse transcriptase were included. All RT-PCR primers are listed in Table 2.

### Protein extraction and western blot analysis

To assess Rim101 processing, overnight cultures were diluted to an optical density of 1 in 25 ml YPD pH 4 and pH 8. Cells were incubated for 1 hour, washed 1x with water, flash frozen on dry ice, and stored at– 80°C until cell harvesting.

For cell wall integrity pathway activation analysis, overnight YPD cultures were diluted to an optical density of 0.8 in 25 ml YPD in duplicate and incubated for 2.5 hours at 30°C. Cultures were then spun down and duplicates were resuspended in YPD or TC medium and incubated at 30°C (YPD) or 37°C (TC). After 3.5 hours, samples were taken, washed 1x with water, flash frozen on dry ice, and stored at -80°C until cell harvesting.

For protein extraction, cells were lysed by bead beating and the lysate was collected in 1.4 ml NP40 lysis buffer (6 mM Na_2_HPO_4_, 4 mM NaH_2_PO_4_, 1% Nonidet P-40, 150 mM NaCl, 2 mM EDTA, 1x protease inhibitors [Complete mini, EDTA-free; Roche], 1x phosphatase inhibitors [Phos-Stop; Roche], and 1 mM phenylmethylsulfonyl fluoride [PMSF]), as described previously [100]. For Western blot analysis, samples were normalized by BCA assay (Thermo Scientific), diluted in 4x lithium dodecyl sulfate (LDS) loading sample buffer, and boiled for 5 minutes. Normalized protein was loaded on a NuPage 4–12% Bis-Tris gel (Invitrogen) and western blots were performed as previously described [100]. To detect Gfp-Rim101, blots were incubated with an anti-GFP primary antibody (1/5,000 dilution; Roche) and an anti-mouse peroxidase-conjugated secondary antibody (1/25,000 dilution; Jackson Labs). To detect phosphorylated Mpk1, blots were incubated with a phospho-p44/42 MAPK (Thr202/Tyr204) rabbit polyclonal primary antibody (1/2,500 dilution; 4370 Cell Signaling Technology) and an anti-rabbit peroxidase-conjugated secondary antibody (1/50,000 dilution; Jackson Labs). Proteins were detected by enhanced chemiluminescence (ECL Prime Western blotting detection reagent; GE Healthcare).

### FM4-64 staining and imaging

Cells from an overnight YPD (30°C) or TC (37°C) culture were normalized and stained with the lipophilic dye, FM4-64 (1/1000 dilution in the indicated medium, Molecular Probes) for 30 minutes shaking, after which the cells were pelleted and YPD or TC medium was refreshed for an additional 30 minutes at with shaking. For microscopy, cells were pelleted, washed 2x with PBS, and resuspended in PBS. FM4-64 was visualized using a Texas Red filter. The same exposure time was used for all images.

### Acid and alkaline secretion analysis

Cells were incubated overnight in phosphate replete minimal medium (15 mM dextrose, 10 mM MgSO_4_, 13 mM glycine, 3 μM thymine, 0.4% KH_2_PO_4_). Cultures were then diluted to an OD of 0.9 in phosphate replete or phosphate deficient minimal medium (15 mM dextrose, 10 mM MgSO_4_, 13 mM glycine, 3 μM thymine, 0.4% KCl) and washed 1x in respective minimal media. 100 μl aliquots were plated in triplicate in a 96-well plate and incubated for 3 hours at 30°C with shaking at 150 rpm. *Para*-Nitrophenylphoshate (pNPP) solutions were prepared by dissolving a 5 mg pNPP substrate tablet (Thermo) in 50 mM sodium acetate (pH 5.2) for acid phosphatase testing or 1x diethanolamine substrate buffer (Thermo) for alkaline phosphatase testing. 100 μl of pNPP solution was added to each well and plates were incubated for 3 hours at 37°C with shaking at 150 rpm. Phosphatase activity was measured at an absorbance of 410 nm and adjusted for cell density, as determined by absorbance at 600 nm, over a time course of 3 hours.

### Transmission electron microscopy

Overnight YPD and TC cultures were diluted to an OD of 0.5 and recovered in the same media for 4–6 hours. Cells were prepared as described previously [14,68,101]. Briefly, cells were spun down and washed 1x in pre-fixation mix (0.1 M sorbitol, 1 mM MgCl_2_, 1 mM CaCl_2_, 2% gluteraldehyde in 0.1 M PIPES, pH 6.8), followed by fixing in fresh pre-fixation mix overnight at 4°C. The next day, cells were washed 3x for 10-minute intervals in water. Next the cells were washed 3x in 2% KMnO_4_, and post-fixed for 45 minutes at room temperature in fresh 2% KMnO_4_. The cells were then washed repeatedly in water until no purple color was visible, and partially dehydrated for 10-minute intervals in increasing concentrations of ethanol (30%, 50%, 70%). Partially dehydrated samples were submitted to the Duke Shared Materials Instrumentation Facility (SMIF) for further processing, embedding, and sectioning as follows: Samples were rinsed thoroughly in PBS and post-fixed in 1% osmium tetroxide for 1 hour at room temperature. Samples were then stained with 0.5% uranyl acetate for 1 hour, further dehydrated in a series of graded ethanol (30%, 50%, 70%, 90%, 100%) and infiltrated overnight in resin. Samples were then embedded in resin and cured in a 55°C oven for 48 hours. The cured samples were thin sectioned with an ultramicrotome to approximately 60–90 nm. Thin-sections were mounted on copper grids and stained with uranyl acetate and lead citrate to enhance contrast. Grids were examined and digital images were taken on the FEI Tecnai G2 Twin transmission electron microscope with an Eagle digital camera.

### Generation of bone marrow derived macrophages and dendritic cells

Bone marrow cells derived from female C57BL/6 mice purchased from Jackson Laboratories were used as WT controls for all experiments, unless otherwise noted. MyD88^-/-^ and TLR2/4^-/-^ bone marrow cells were a generous gift from Marcel Wüthrich at the University of Wisconsin-Madison. Bone marrow cells from Card9^-/-^ mice were provided by Floyd Wormley. Dectin1^-/-^ mice were a generous gift from Mari Shinohara at Duke University. C3H/HeOuJ TLR4 mutant mice (Stock no. 000659), C3H/HeJ control mice (Stock no. 000635), and TLR2^-/-^ mice (Stock no. 004650, [102]) were purchased from Jackson Laboratories.

Murine bone marrow cells were isolated and prepared as previously described [16,103,104]. Briefly, femurs and tibias were isolated from mice and each bone was flushed with 5 to 10 ml cold PBS using a 27½ gauge needle. Red blood cells were lysed in 1x RBC lysis buffer (0.15 M NH_4_Cl, 1 mM NaHCO_3_, pH 7.4) and cells were resuspended in 1x Dulbecco’s modified Eagle’s medium (DMEM; + 4.5 g/L D-Glucose, + L-Glutamine, +110 mg/L sodium pyruvate) with 1 U/ml pencillin/streptomycin. Bone marrow cells were cryopreserved in 90% FBS/10% endotoxin-free DMSO at a concentration of 1 x 10^7^ cells/ml and later thawed for use as previously described [103].

Fresh or frozen bone marrow cells were used to generate bone marrow derived macrophages (BMMs) or bone marrow derived dendritic cells (BMDCs). BMMs were differentiated in BMM medium (1x Dulbecco’s modified Eagle’s medium [DMEM; + 4.5 g/L D-Glucose, + L-Glutamine, +110 mg/L sodium pyruvate], 10% fetal bovine serum [FBS; non-heat inactivated], 1 U/ml penicillin/streptomycin) with 3 ng/ml recombinant mouse GM-CSF (rGM-CSF; R&D Systems or BioLegend) at a concentration of 2.5 x 10^5^ cells/ml in 150 x 15 mm petri plates at 37°C with 5% CO_2_. The media was refreshed after 3–4 days and the cells were harvested on day 7 as previously described [103]. BMMs were counted (by hemocytometer, with Trypan blue to discount dead cells), plated in BMM medium in 96-well plates at a concentration of 5 x 10^4^ cells/well, and incubated at 37°C with 5% CO_2_ overnight prior to fungal co-culture experiments.

BMDCs were differentiated in BMDC medium (1x RPMI, 10% FBS [non-heat inactivated], 1 U/ml penicillin/streptomycin, 1x beta-mercaptoethanol) with 20 ng/ml rGM-CSF at a concentration of 5 x 10^6^ cells/ml in 20 ml in 150 x 15 mm petri plates at 37°C with 5% CO_2_. After 3 days an additional 20 ml of BMDC medium with 20 ng/ml rGM-CSF was added to plates. After 6 days, 20 ml of culture supernatant was collected, centrifuged, resuspended in fresh BMDC medium with rGM-CSF and returned to the culture plate. BMDCs were harvested on day 10 as described previously [105] and BMDCs were counted, plated in BMDC medium in 96-well plates at a concentration of 5 x 10^4^ cells/well, and incubated at 37°C with 5% CO_2_ overnight prior to fungal co-culture experiments.

### *In vitro* fungal co-culture experiments

BMM and BMDC co-cultures with *C*. *neoformans* were performed as described previously [16]. Briefly, C. *neoformans* cells were washed 2x with PBS, counted, and added to BMM or BMDC containing 96-well plates at a concentration of 5 x 10^5^ fungal cells per well (10:1 *C*. *neoformans* cells:BMMs/BMDCs). Isolated cell wall material was added at a concentration of 10 mg/ml. Co-cultures were incubated for the indicated amount of time at 37°C with 5% CO_2_. Supernatants were collected and stored at -80°C until analysis. Secreted cytokines (TNF-α) were quantified in supernatants by enzyme-linked immunosorbent assay (ELISA; BioLegend). Data are represented as the average TNF-α values (pg/ml) for biological replicates; each fungal strain was tested a minimum of 3 times. BMM/BMDC only control wells, in which fresh media was added in lieu of fungi are included as negative controls. Ultrapure lipopolysaccharide (List Biolabs) and zymosan from *S*. *cerevisiae* (Sigma) were diluted to the indicated concentrations in BMM medium and used as positive controls.

As described previously [16], the *cap59Δ* mutation causes cell aggregation that makes quantification by hemocytometer inaccurate. As a result, these strains were normalized to 2 mg wet cell pellet/ml of medium, which was used previously for other mutants with similar mass/cell ratios and approximates the milligram-per-milliliter concentration used for standard co-culture experiments [16].

### Animal experiments

We used the murine inhalation model of Cryptococcosis to assess virulence [45]. For each strain, 9–10 female C57BL/6 mice and 9–10 male and female BALB/c mice were used. Mice were anesthetized by isoflurane inhalation and intranasally inoculated with 1 x 10^5^ fungal cells of the following strains: WT (H99), *mar1Δ* (MAK1), and *mar1Δ + MAR1* (MAK11). Mice were monitored over the course of 40 days and sacrificed based on clinical endpoints that predict mortality. The statistical significance of difference between survival curves of mice infected with different strains was determined by log-rank test with Bonferroni correction (GraphPad Prism).

An additional 9–10 mice per strain were intranasally inoculated as described above for organ burden. Mice (5 per time point) were sacrificed on days 1 and 4 post inoculation. From each mouse 1 lung was harvested, weighed, and homogenized in cold PBS. Colony forming units (CFU) were calculated by quantitative culture and are represented as CFU/gram of tissue. For BALB/c post-mortem CFU analysis, lungs and brains were harvested from mice and homogenized in 1 ml PBS. Viable cells were calculated by quantitative culture and are represented as CFU/ml.

### Ethics statement

All animal experiments were performed in compliance with guidelines at Duke University, the University of Texas at San Antonio, and the American Veterinary Medical Association. All mice were anesthetized by isoflurane inhalation. Mice were sacrificed by CO_2_ with approved secondary methods of ensuring animal death. The Duke University Institutional Animal Care and Use Committee reviewed and approved the protocol (A138-17-06) used for animal experimentation in these studies. The specific projects were reviewed for congruence with this protocol, and approval was granted on 6/29/2015. Duke University maintains an animal program that is registered with the United States Department of Agriculture (Animal Welfare Act, Customer Number: 863), assured through the National Institutes of Health/Public Health Service (Assurance Number D16-00123 (A3195-01)), and accredited with AAALAC International (Accreditation Number: 363).

## Supporting information

S1 Fig Cell wall staining in additional conditions. (A) WT cells incubated and stained as described in Fig 3. Bar, 10 μM. (B) Cell wall staining was assessed after incubation in YPD buffered to pH 8 or TC medium supplemented with 1x complete amino acids for 16–18 hours at 30°C with shaking. Cells were stained with FITC-conjugated WGA and imaged by fluorescent microscopy with the GFP filter.
(TIF)

S2 Fig Mar1 is not regulated by or regulating the Rim pathway. (A) *MAR1* expression is induced in TC medium. WT cells were incubated for 1.5 hours in YPD (30°C) or TC (37°C), followed by RNA extraction and cDNA synthesis. Expression of *MAR1* was determined by real-time PCR with fold change calculated relative to WT YPD levels and normalized to the expression of an internal control. Data represent means of results from 3 independent *C*. *neoformans* cultures and RNA extractions per condition. **, p = 0.0017 as determined by unpaired t-test. (B) Rim101 processing is intact in *mar1Δ* cells. WT and *mar1Δ* cells were incubated for 1 hour at the indicated pH, followed by western blotting using an α-GFP antibody.
(TIF)

S3 Fig Mar1 is not a direct member of the cell wall integrity (CWI) pathway. (A) CWI pathway signaling is intact in *mar1Δ* cells. WT and *mar1Δ* cells were incubated overnight in YPD and refreshed in YPD (30°C) or TC (37°C) for 3.5 hours, followed by western blotting using an α-phospho-Mpk1 antibody. Left panel is a representative blot image; Right panel is quantification of bands from 3 replicate experiments using ImageJ/Fiji software. (B) *mar1Δ* and *mpk1Δ* have combined effects on WGA staining. WT, *mar1Δ*, *mpk1Δ*, and *mar1Δ mpk1Δ* double mutant cells were incubated for 16–18 hours in YPD (30°C) or TC (30°C) followed by staining with WGA. Live cells were imaged by fluorescent microscopy and average fluorescence was quantified for at least 100 cells using ImageJ/Fiji software.
(TIF)

S4 Fig Additional β-(1,3)-glucan synthase localization images. Live cells were imaged using DeltaVision deconvolution fluorescent microscopy with the GFP filter. Images were deconvolved using softWoRx software. (A) Fks1-Gfp localization is similar in WT and *mar1Δ* mutant strains after incubation in YPD medium. Cells were incubated for 16–18 hours in YPD at 30°C prior to imaging. Bar, 10 μM. (B) Localization of Fks1-Gfp to the plasma membrane after incubation in TC media is decreased in an independent *mar1Δ* mutant. Cells were incubated for 16–18 in TC medium at 37°C prior to imaging. Bar, 10 μM.
(TIF)

S5 Fig Macrophage activation by *mar1Δ* is independent of capsule. Cultures of *cap59Δ* and *mar1Δ cap59Δ* were incubated for 16–18 hours in TC medium at 37°C. 2 mg/ml wet weight of each strain was co-cultured with BMMs for 6 hours, followed by quantification of TNF-α (pg/ml) in the supernatant by ELISA. Data represent 3 replicates from 3 independent experiments. **, p < 0.01 *mar1Δ cap59Δ* vs. *cap59Δ* as determined by one-way ANOVA with Tukey’s multiple comparisons test.
(TIF)

S6 Fig C3H/HeJ and C3H/HeOuJ BMMs respond normally to control ligands. BMMs were harvested from the indicated mouse strains and co-incubated with 10 ng/ml LPS or 10 μg/ml zymosan for 6 hours, followed by quantification of TNF-α (pg/ml) in the supernatant by ELISA. Data represent means of 3 replicates from 2 independent experiments (n = 6).
(TIF)
