# Supplementary figures and images for: Defects in intracellular trafficking of fungal cell wall synthases lead to aberrant host immune recognition
Source: PLoS Pathog. 2018 Jun 4;14(6):e1007126. doi: 10.1371/journal.ppat.1007126 (PMC6002136; doi:10.1371/journal.ppat.1007126)

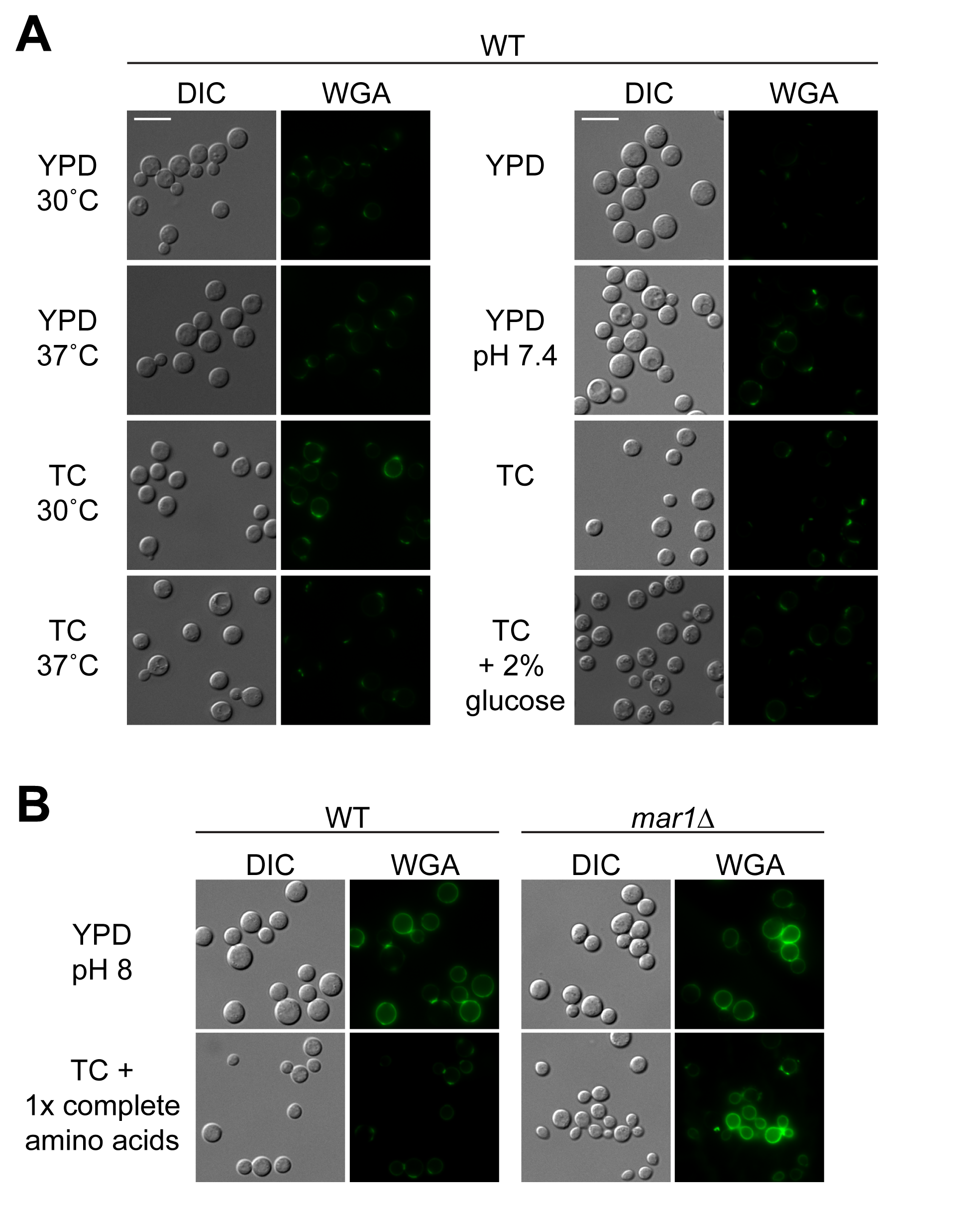

Supplement: S1 Fig — (A) WT cells incubated and stained as described in Fig 3. Bar, 10 μM. (B) Cell wall staining was assessed after incubation in YPD buffered to pH 8 or TC medium supplemented with 1x complete amino acids for 16–18 hours at 30°C with shaking. Cells were stained with FITC-conjugated WGA and imaged by fluorescent microscopy with the GFP filter. (TIF) [file ppat.1007126.s001.tif]

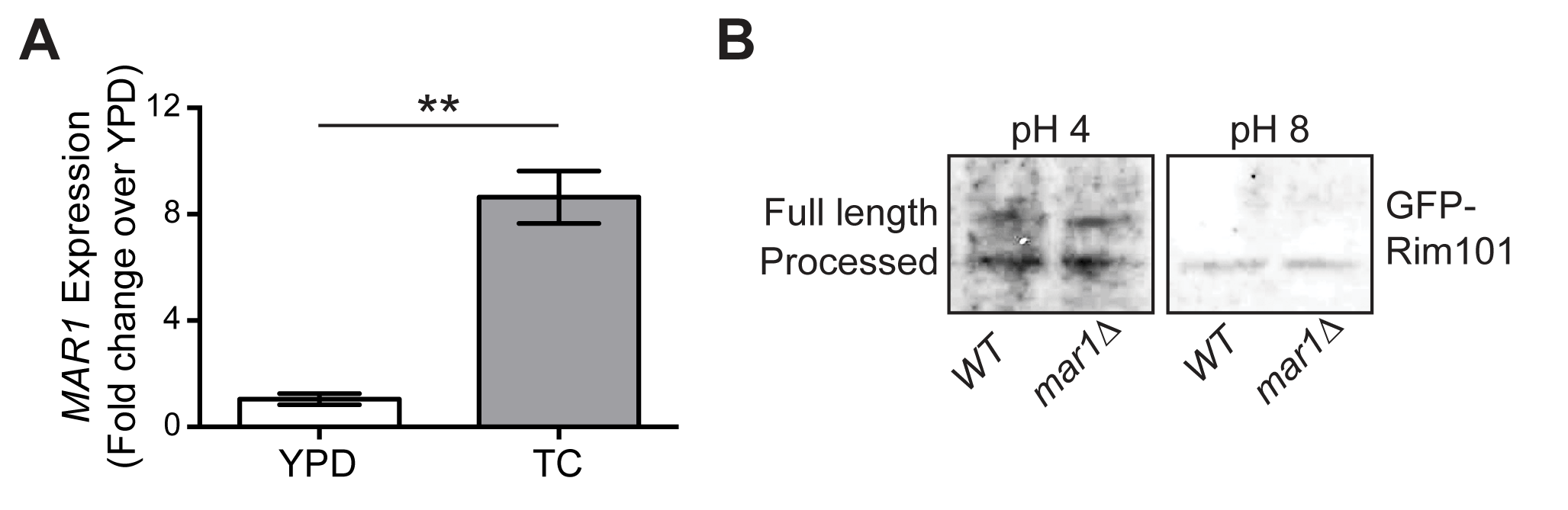

Supplement: S2 Fig — (A) MAR1 expression is induced in TC medium. WT cells were incubated for 1.5 hours in YPD (30°C) or TC (37°C), followed by RNA extraction and cDNA synthesis. Expression of MAR1 was determined by real-time PCR with fold change calculated relative to WT YPD levels and normalized to the expression of an internal control. Data represent means of results from 3 independent C. neoformans cultures and RNA extractions per condition. **, p = 0.0017 as determined by unpaired t-test. (B) Rim101 processing is intact in mar1Δ cells. WT and mar1Δ cells were incubated for 1 hour at the indicated pH, followed by western blotting using an α-GFP antibody. (TIF) [file ppat.1007126.s002.tif]

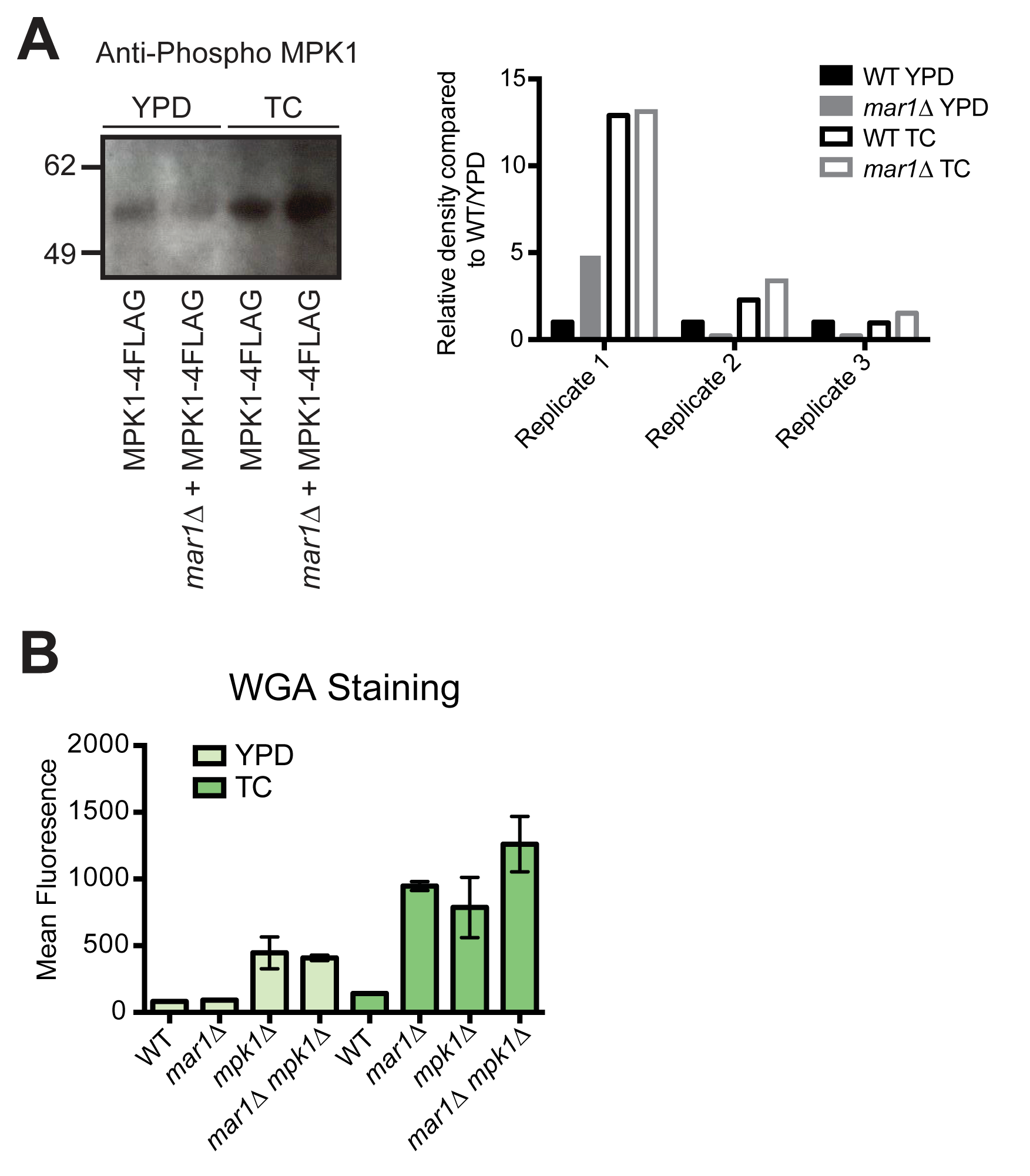

Supplement: S3 Fig — (A) CWI pathway signaling is intact in mar1Δ cells. WT and mar1Δ cells were incubated overnight in YPD and refreshed in YPD (30°C) or TC (37°C) for 3.5 hours, followed by western blotting using an α-phospho-Mpk1 antibody. Left panel is a representative blot image; Right panel is quantification of bands from 3 replicate experiments using ImageJ/Fiji software. (B) mar1Δ and mpk1Δ have combined effects on WGA staining. WT, mar1Δ, mpk1Δ, and mar1Δ mpk1Δ double mutant cells were incubated for 16–18 hours in YPD (30°C) or TC (30°C) followed by staining with WGA. Live cells were imaged by fluorescent microscopy and average fluorescence was quantified for at least 100 cells using ImageJ/Fiji software. (TIF) [file ppat.1007126.s003.tif]

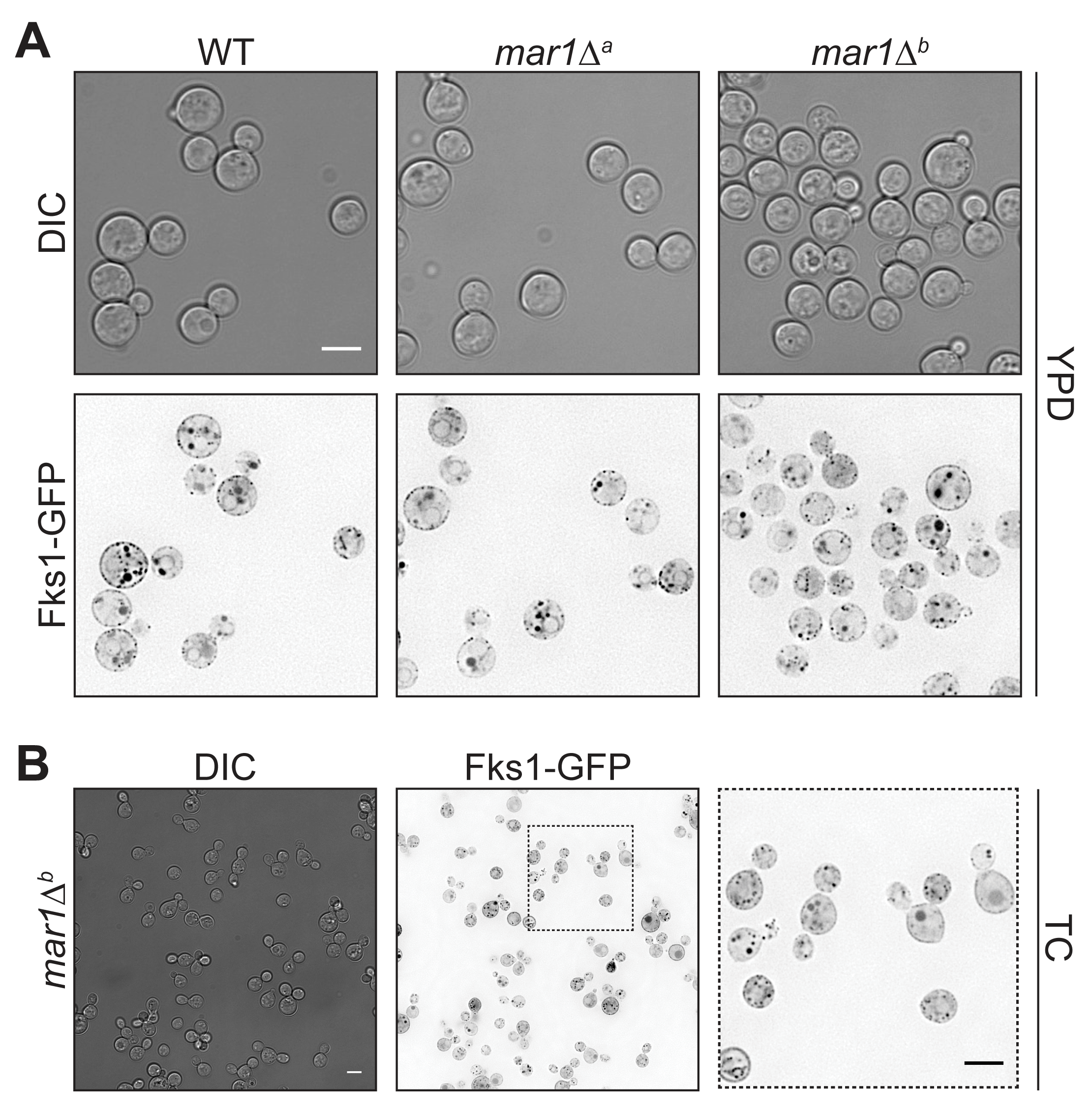

Supplement: S4 Fig — Live cells were imaged using DeltaVision deconvolution fluorescent microscopy with the GFP filter. Images were deconvolved using softWoRx software. (A) Fks1-Gfp localization is similar in WT and mar1Δ mutant strains after incubation in YPD medium. Cells were incubated for 16–18 hours in YPD at 30°C prior to imaging. Bar, 10 μM. (B) Localization of Fks1-Gfp to the plasma membrane after incubation in TC media is decreased in an independent mar1Δ mutant. Cells were incubated for 16–18 in TC medium at 37°C prior to imaging. Bar, 10 μM. (TIF) [file ppat.1007126.s004.tif]

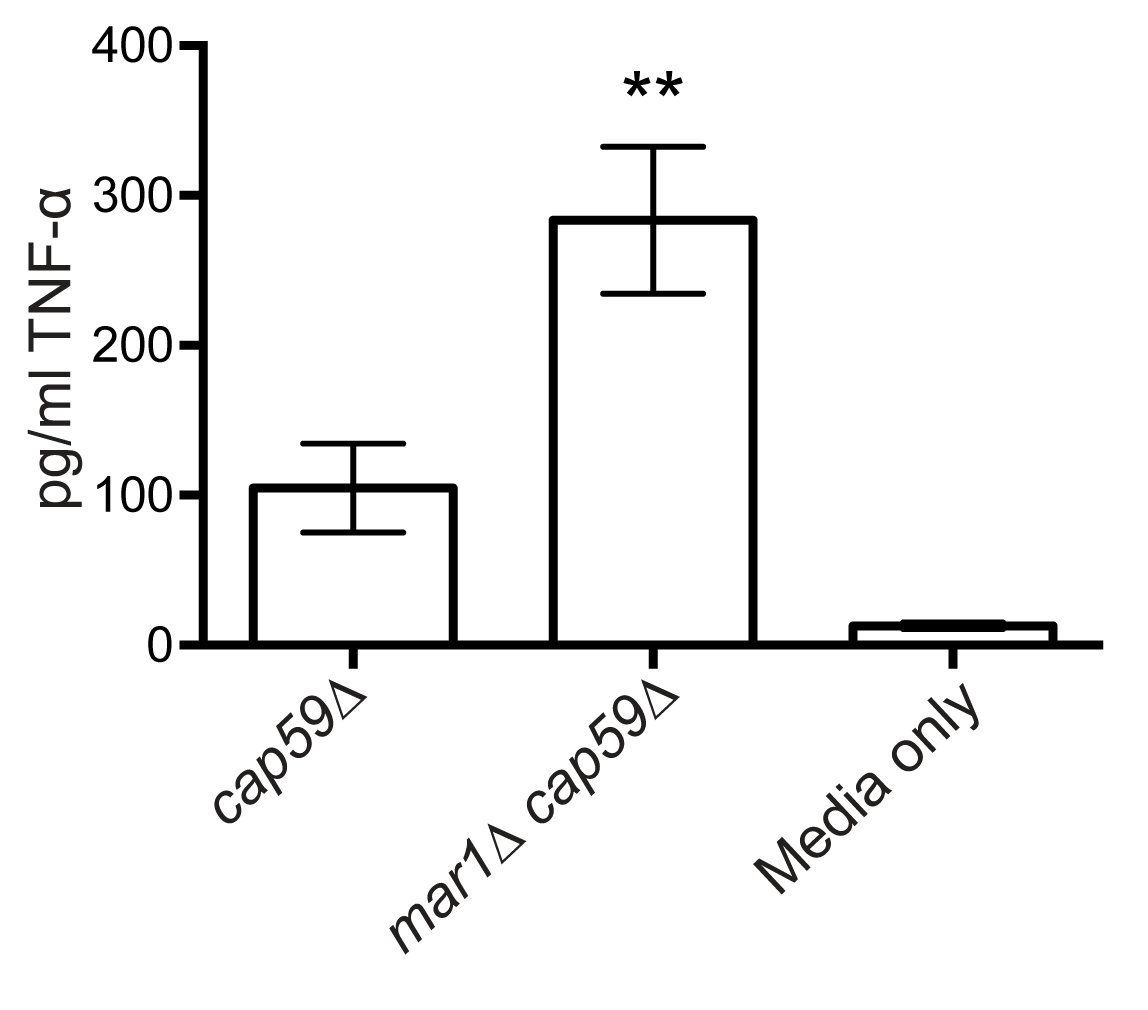

Supplement: S5 Fig — Cultures of cap59Δ and mar1Δ cap59Δ were incubated for 16–18 hours in TC medium at 37°C. 2 mg/ml wet weight of each strain was co-cultured with BMMs for 6 hours, followed by quantification of TNF-α (pg/ml) in the supernatant by ELISA. Data represent 3 replicates from 3 independent experiments. **, p < 0.01 mar1Δ cap59Δ vs. cap59Δ as determined by one-way ANOVA with Tukey’s multiple comparisons test. (TIF) [file ppat.1007126.s005.tif]

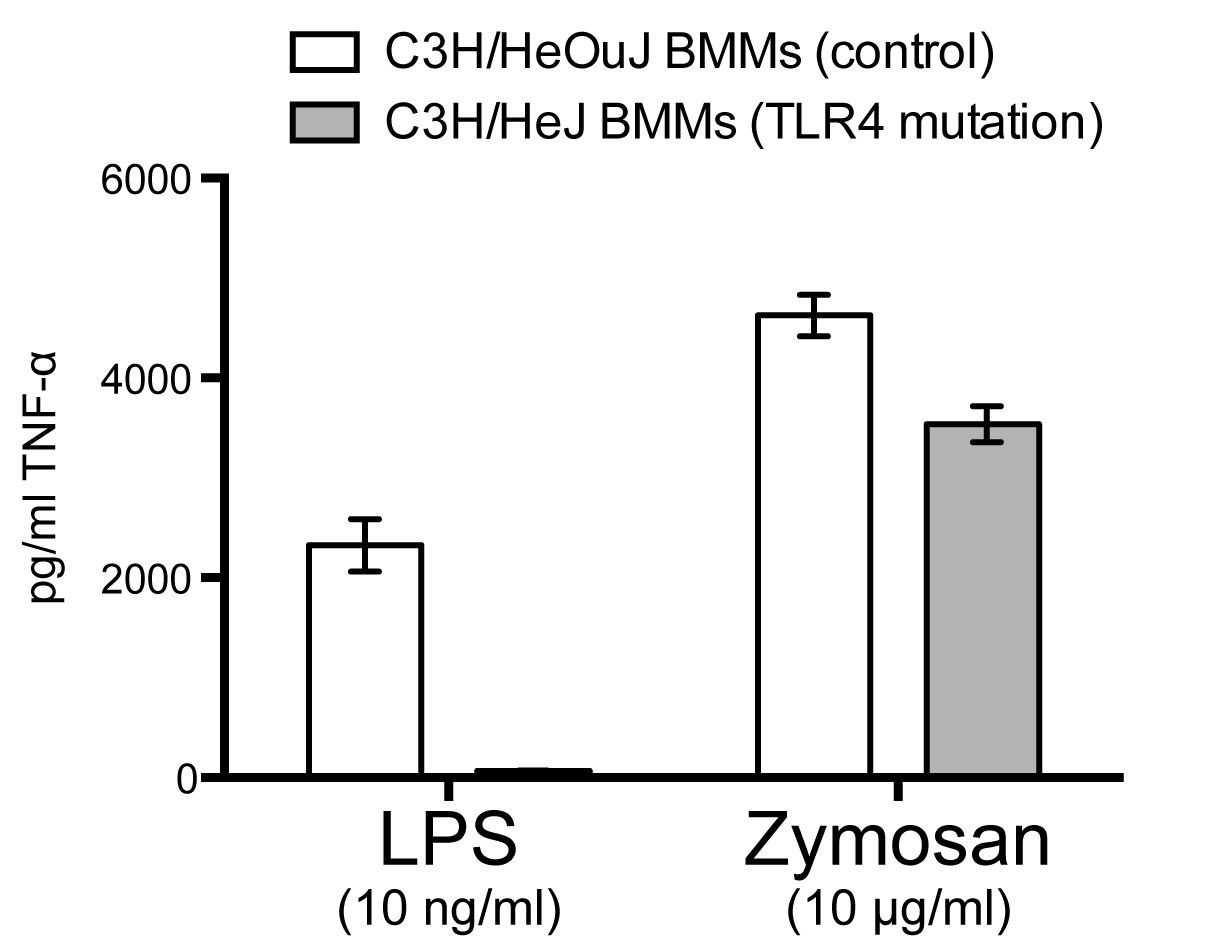

Supplement: S6 Fig — BMMs were harvested from the indicated mouse strains and co-incubated with 10 ng/ml LPS or 10 μg/ml zymosan for 6 hours, followed by quantification of TNF-α (pg/ml) in the supernatant by ELISA. Data represent means of 3 replicates from 2 independent experiments (n = 6). (TIF) [file ppat.1007126.s006.tif]
